# Supplementary material for: BRCA1 binds TERRA RNA and suppresses R-Loop-based telomeric DNA damage
Source: Nat Commun. 2021 Jun 10;12:3542. doi: 10.1038/s41467-021-23716-6 (PMC8192922; doi:10.1038/s41467-021-23716-6)
Supplement: Supplementary file 1 — Supplementary Information [file 41467_2021_23716_MOESM1_ESM.pdf]

SUPPLEMENTARY INFORMATION

BRCA1 binds TERRA RNA and suppresses R-Loop-based telomeric DNA damage

Jekaterina Vohhodina[1,2*], Liana J. Goehring[1], Ben Liu[1,2], Qing Kong[1,2], Vladimir V. Botchkarev Jr.[1,2], Mai Huynh[1], Zhiqi Liu[1], Fieda O. Abderazzaq[1,2], Allison P. Clark[1,2], Scott B. Ficarro[1,3,4,5], Jarrod A. Marto[1,3,4,5], Elodie Hatchi[1,2], and David M. Livingston[1,2*]

[1] Department of Cancer Biology, Dana-Farber Cancer Institute, 450 Brookline Avenue, Boston, MA 02215, USA

[2] Department of Genetics, Harvard Medical School, 25 Shattuck Street, Boston, MA 02115, USA

[3] Blais Proteomics Center, Dana-Farber Cancer Institute, 450 Brookline Avenue, Boston, MA 02215, USA

[4] Department of Oncologic Pathology, Dana-Farber Cancer Institute, 450 Brookline Avenue, Boston, MA 02215, USA

[5] Department of Pathology, Brigham and Women's Hospital, Harvard Medical School, 75 Francis street, Boston, MA 02115, USA

*Correspondence: jekaterina_vohhodina@dfci.harvard.edu (JV), david_livingston@dfci.harvard.edu (DML)

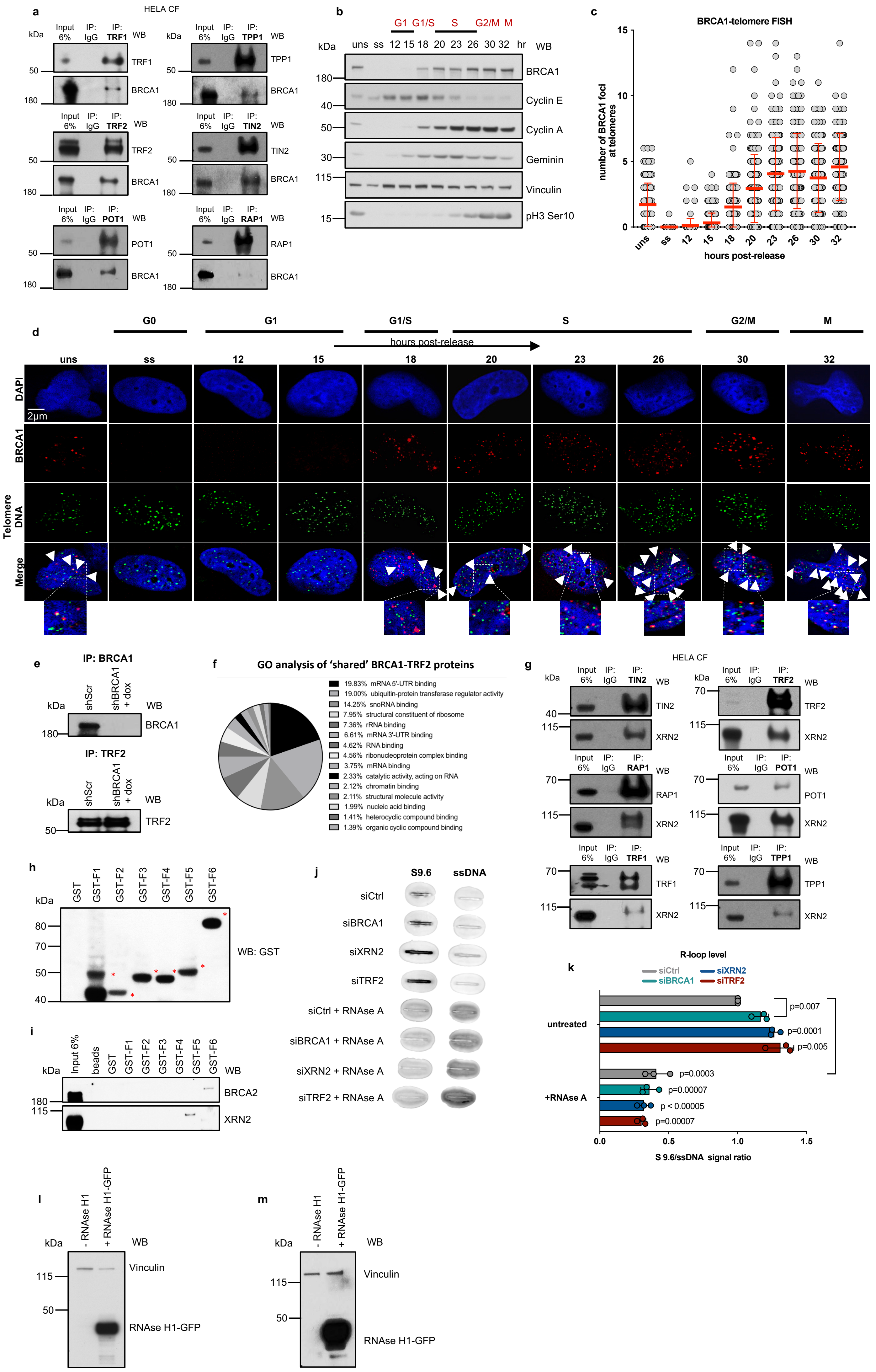

a
HELA CF
Input 6%
IP: IgG
IP: TRF1
IP: TPP1
IP: TRF2
IP: TIN2
IP: POT1
IP: RAP1
WB
kDa
TRF1
BRCA1
TPP1
TRF2
TIN2
POT1
RAP1
b
G1 G1/S S G2/M M
uns ss 12 15 18 20 23 26 30 32 hr
BRCA1
Cyclin E
Cyclin A
Geminin
Vinculin
pH3 Ser10
c
BRCA1-telomere FISH
number of BRCA1 foci at telomeres
hours post-release
d
G0 G1 G1/S S G2/M M
hours post-release
uns ss 12 15 18 20 23 26 30 32
DAPI
BRCA1
Telomere DNA
Merge
2μm
e
IP: BRCA1
shScr
shBRCA1 + dox
IP: TRF2
f
GO analysis of 'shared' BRCA1-TRF2 proteins
19.83% mRNA 5'-UTR binding
19.00% ubiquitin-protein transferase regulator activity
14.25% snoRNA binding
7.95% structural constituent of ribosome
7.36% rRNA binding
6.61% mRNA 3'-UTR binding
4.62% RNA binding
4.56% ribonucleoprotein complex binding
3.75% mRNA binding
2.33% catalytic activity, acting on RNA
2.12% chromatin binding
2.11% structural molecule activity
1.99% nucleic acid binding
1.41% heterocyclic compound binding
1.39% organic cyclic compound binding
g
HELA CF
XRN2
h
GST GST-F1 GST-F2 GST-F3 GST-F4 GST-F5 GST-F6
WB: GST
i
Input 6% beads GST GST-F1 GST-F2 GST-F3 GST-F4 GST-F5 GST-F6
BRCA2
XRN2
j
S9.6 ssDNA
siCtrl
siBRCA1
siXRN2
siTRF2
siCtrl + RNAse A
siBRCA1 + RNAse A
siXRN2 + RNAse A
siTRF2 + RNAse A
k
R-loop level
siCtrl siXRN2
siBRCA1 siTRF2
untreated
+RNAse A
p=0.007
p=0.0001
p=0.005
p=0.0003
p=0.00007
p < 0.00005
p=0.00007
S 9.6/ssDNA signal ratio
l
- RNAse H1
+ RNAse H1-GFP
Vinculin
RNAse H1-GFP
m
- RNAse H1
+ RNAse H1-GFP
Vinculin
RNAse H1-GFP

Supplementary Figure 1. BRCA1 associates with the shelterin proteins and XRN2 in an R-loop-dependent manner at telomeres

a. Co-IP assays demonstrating endogenous interactions of the shelterin proteins with BRCA1 in HeLa CFs. The immune complexes were analyzed by immunoblotting with the indicated antibodies. n=5 independent experiments. **b.** Immunoblot of T98G cells confirming synchronization efficiency with cell cycle specific markers: cyclin A, cyclin E, geminin, $pH3^{Ser10}$, and BRCA1. n=3 independent experiments. **c**. Quantitation of BRCA1-telomeric DNA co-localized foci in synchronized T98G cells. Mean values from three independent experiments are represented by red lines, ±SD. At least n=50 cells were counted per experiment. **d**. Immunofluorescent staining of the BRCA1 protein (shown in red) combined with telomere DNA-FISH (shown in green) using a CCCTAA-FAM probe, confirming co-localization events of BRCA1 at telomeres in synchronized T98G cells (see arrowheads). n=3 independent experiments. **e.** Representative aliquots of the pulldown assays of BRCA1 and TRF2 used for nanoLC-MS analysis. CFs of HME cells were immunoprecipitated with BRCA1 and TRF2 antibodies, respectively, n=3 independent experiments. **f.** Functional annotation of the proteins found to be associated with both BRCA1 and TRF2 using PANTHER (v.14). **g.** Co-IP assays demonstrating endogenous interactions of the shelterin proteins with XRN2 in CFs of HeLa cells. n=5 independent experiments. **h.** Immunoblot of GST fusion proteins (detected by GST antibody) used in **i** revealing their relative abundance. n=3 independent experiments. **i**. The interaction between BRCA1 and XRN2 *in vitro*. GST-BRCA1 polypeptide fragments were incubated with HeLa cell extract, and the immune complexes were analyzed by immunoblotting using XRN2 antibody. BRCA2 binding to GST-F6 was used as a positive control. n=3 independent experiments. **j.** Levels of R-loops in control and BRCA1-, XRN2-, or TRF2-depleted cells detected by slot blot analysis, using S9.6 antibody and ssDNA as a normalization control. **k**. Quantitation of R-loop amounts as depicted in **j.** The mean intensity of the S9.6 fluorescent signal, normalized to the ssDNA signal, is plotted, n=3 independent experiments, ±SD. p-values were obtained using a two-tailed Student's t-test. **l-m.** Immunoblots confirming expression of the GFP-tagged RNAse H1-expressing vector in HeLa (**l**) and U2OS (**m**) cells. n=3 independent experiments. Source data are provided as a Source data file.

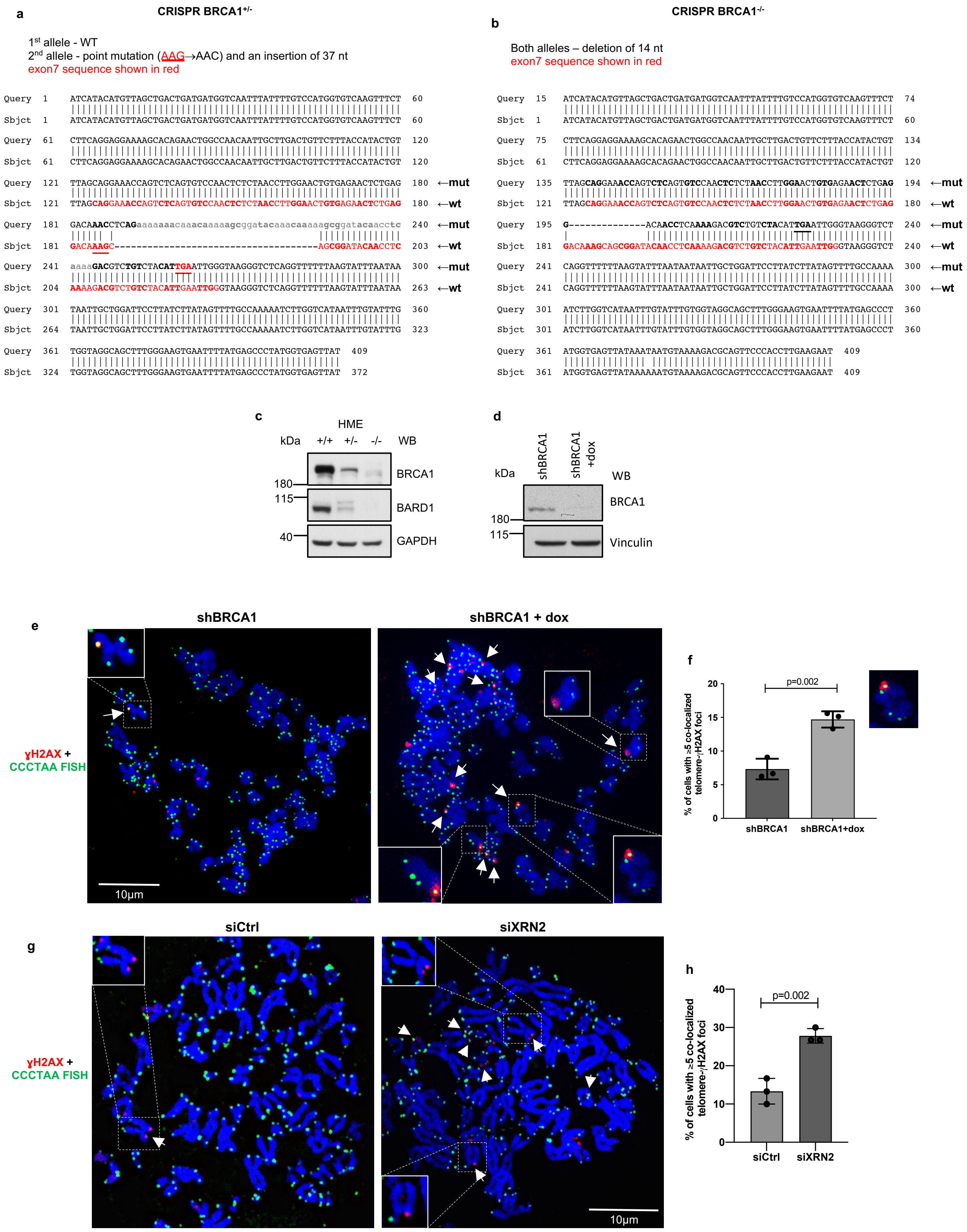
a
CRISPR BRCA1+/-
1st allele - WT
2nd allele - point mutation (AAG→AAC) and an insertion of 37 nt
exon7 sequence shown in red
←mut
←wt
b
CRISPR BRCA1-/-
Both alleles – deletion of 14 nt
exon7 sequence shown in red
c
HME
kDa
+/+
+/-
-/-
WB
BRCA1
BARD1
GAPDH
180
115
40
d
shBRCA1
shBRCA1 +dox
kDa
WB
BRCA1
Vinculin
180
115
e
shBRCA1
shBRCA1 + dox
γH2AX +
CCCTAA FISH
10μm
f
p=0.002
% of cells with ≥5 co-localized telomere-γH2AX foci
shBRCA1
shBRCA1+dox
g
siCtrl
siXRN2
γH2AX +
CCCTAA FISH
10μm
h
p=0.002
% of cells with ≥5 co-localized telomere-γH2AX foci
siCtrl
siXRN2

Supplementary Figure 2. BRCA1 and XRN2 suppress DNA damage at telomeres
a-b. Sequence alignment of $BRCA1^{+/+}$ vs $BRCA1^{+/-}$ and $BRCA1^{-/-}$ CRISPR HME clones. **a**. HME $BRCA1^{+/-}$ clone: one allele is WT, the 2nd allele has a point mutation (AAG→AAC, underlined) and an insertion of 37 nt. **b**. HME $BRCA1^{-/-}$ clone: contains a deletion of 14 nt from both alleles. The BRCA1 exon 7 sequence with the CRISPR targeted region is marked in red. **c.** Immunoblot of isogenic BRCA1 parental ($^{+/+}$), heterozygous ($^{+/-}$), and knockout ($^{-/-}$) CRISPR HME clones, documenting their BRCA1 and BARD1 expression status. n=10 independent experiments. **d.** Immunoblot of HME cells carrying a doxycycline-inducible shBRCA1 gene, confirming BRCA1 depletion. Cells were exposed to doxycycline to activate BRCA1 depletion. n=5 independent experiments. **e.** Representative microscopic images of cytospun metaphase spreads from control and BRCA1-depleted HME cells, which were stained with an antibody directed at γH2AX (red) and then for telomeric DNA (green) using a CCCTAA-FAM probe. Chromosome ends displaying a γH2AX signal are identified by arrows. n=3 independent experiments. **f.** Mean percentage of control and BRCA1-depleted HME cells with ≥5 γH2AX-telomere DNA co-localization regions from three independent experiments. At least n=30 chromosome spreads scored/experiment, ±SD. The statistical significance of the data was assessed using a two-tailed Student's t-test. **g-h.** Same as in **e-f**, only a comparison of control and XRN2-depleted HME cells was undertaken from three independent experiments. At least n=30 chromosome spreads scored/experiment, ±SD. The statistical significance of the data was assessed using a two-tailed Student's t-test. Source data are provided as a Source data file.

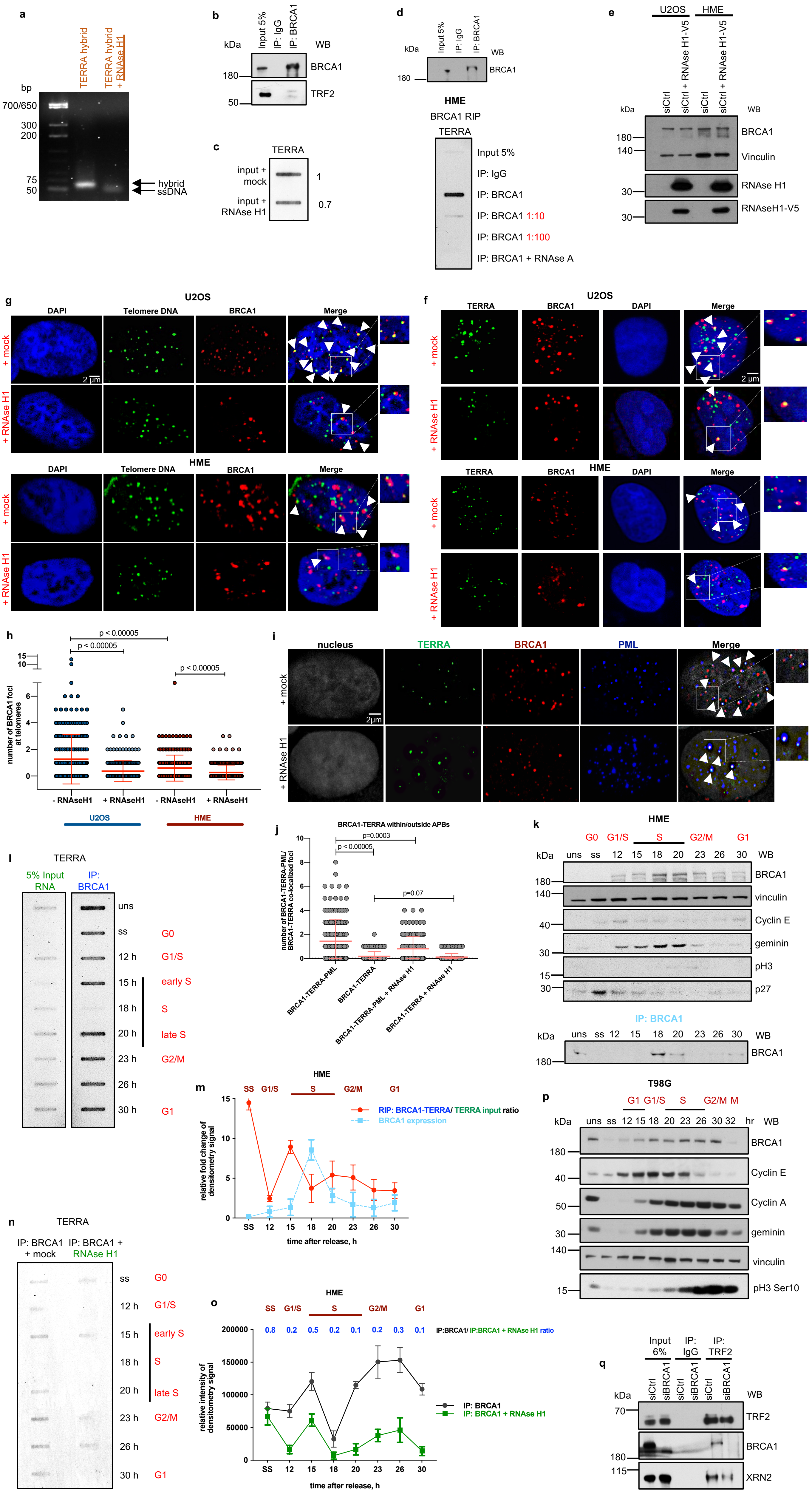

a
TERRA hybrid
TERRA hybrid + RNAse H1
bp
700/650
300
200
75
50
hybrid
ssDNA
b
kDa
Input 5%
IP: IgG
IP: BRCA1
WB
BRCA1
TRF2
180
50
c
TERRA
input + mock
input + RNAse H1
1
0.7
d
kDa
Input 5%
IP: IgG
IP: BRCA1
WB
BRCA1
180
HME
BRCA1 RIP
TERRA
Input 5%
IP: IgG
IP: BRCA1
IP: BRCA1 1:10
IP: BRCA1 1:100
IP: BRCA1 + RNAse A
e
U2OS
HME
siCtrl
siCtrl + RNAse H1-V5
kDa
WB
BRCA1
Vinculin
RNAse H1
RNAseH1-V5
g
U2OS
DAPI
Telomere DNA
BRCA1
Merge
+ mock
+ RNAse H1
2 µm
HME
f
TERRA
h
p < 0.00005
number of BRCA1 foci at telomeres
- RNAseH1
+ RNAseH1
U2OS
HME
i
nucleus
PML
2µm
j
BRCA1-TERRA within/outside APBs
p=0.0003
p=0.07
number of BRCA1-TERRA-PML/ BRCA1-TERRA co-localized foci
BRCA1-TERRA-PML
BRCA1-TERRA
BRCA1-TERRA-PML + RNAse H1
BRCA1-TERRA + RNAse H1
k
HME
G0
G1/S
S
G2/M
G1
uns
ss
vinculin
Cyclin E
geminin
pH3
p27
IP: BRCA1
l
5% Input RNA
IP: BRCA1
12 h
15 h
18 h
20 h
23 h
26 h
30 h
early S
late S
m
RIP: BRCA1-TERRA/ TERRA input ratio
BRCA1 expression
relative fold change of densitometry signal
time after release, h
n
IP: BRCA1 + mock
IP: BRCA1 + RNAse H1
o
0.8
0.2
0.5
0.2
0.1
0.2
0.3
0.1
IP:BRCA1/ IP:BRCA1 + RNAse H1 ratio
IP: BRCA1
IP: BRCA1 + RNAse H1
relative intensity of densitometry signal
p
T98G
G1
M
hr
Cyclin A
pH3 Ser10
q
Input 6%
IP: IgG
IP: TRF2
siCtrl
siBRCA1
XRN2

Supplementary Figure 3. BRCA1-TERRA interactions depend upon cell cycle and R-loop formation

a. Integrity of TERRA hybrids was confirmed using RNase H1 which specifically digested RNA in the hybrids, leaving ssDNA as a product. n=3 independent experiments. **b.** Immunoblot of an aliquot of the BRCA1 RIP assay in **Fig**. **2e**. n=3 independent experiments. **c.** Slot blot analysis of TERRA expression in -/+ RNAse H1-treated cells employed for the BRCA1 RIP assay in **Fig**. **2e**. n=3 independent experiments. **d.** RIP assay in HME cells. The immunoblot represents an aliquot of a BRCA1 RIP. DNAse-treated IP'd RNA was subjected to slot blot analysis with a CCCTAA-DIG probe. n=3 independent experiments. **e.** An immunoblot confirming the transfection efficiency of a V5-tagged RNAse H1-expressing vector in HME and U2OS cells. n=3 independent experiments. **f.** Representative images of IF-RNA FISH assessing BRCA1-TERRA co-localization in mock-, or RNAse H1-treated U2OS and HME cells in **Fig. 2f.** n=3 independent experiments. **g-h.** BRCA1-telomere DNA co-localization and respective quantitation in U2OS and HME cells from three independent experiments. At least n=60 cells scored/experiment, ±SD. p-values were obtained using a two-tailed Student's t-test. **i-j.** IF-RNA FISH and respective quantitation of TERRA-BRCA1-PML co-localization in mock-, or RNAse H1-treated U2OS cells from three independent experiments. At least n=50 cells scored/experiment, ±SD. p-values were obtained using a two-tailed Student's t-test. **k.** Immunoblotting of HME cells confirming the efficiencies of synchronization and the BRCA1 RIP assay. The p27 expression level served as a serum-starvation (ss) state control. n=3 independent experiments. **l.** The BRCA1 RIP-slot blot analysis performed in synchronized HME. DNAse-treated RNA, isolated from each BRCA1 IP, was used in subsequent TERRA slot blot analysis. **m.** Densitometry analysis of the BRCA1 RIP experiment in **l**, representing the mean ratio of relative fold changes of TERRA RIP values over TERRA input values. Relative BRCA1 expression as depicted in **k**, is shown in blue, n=3 independent experiments, ±SD. **n.** Same as in **l**, only in synchronized mock-, or RNAse H1-treated HME cells. **o.** Densitometry analysis of the BRCA1 RIP experiment in **n**, representing the mean TERRA RIP values -/+ RNAse H1, n=3 independent experiments, ±SD. **p**. Immunoblotting of synchronized T98G cells used for the TRF2 IP in **Fig**. **2j**. n=3 independent experiments. **q.** TRF2 pulldown in control and BRCA1-depleted cells. n=3 independent experiments. Source data are provided as a Source data file.

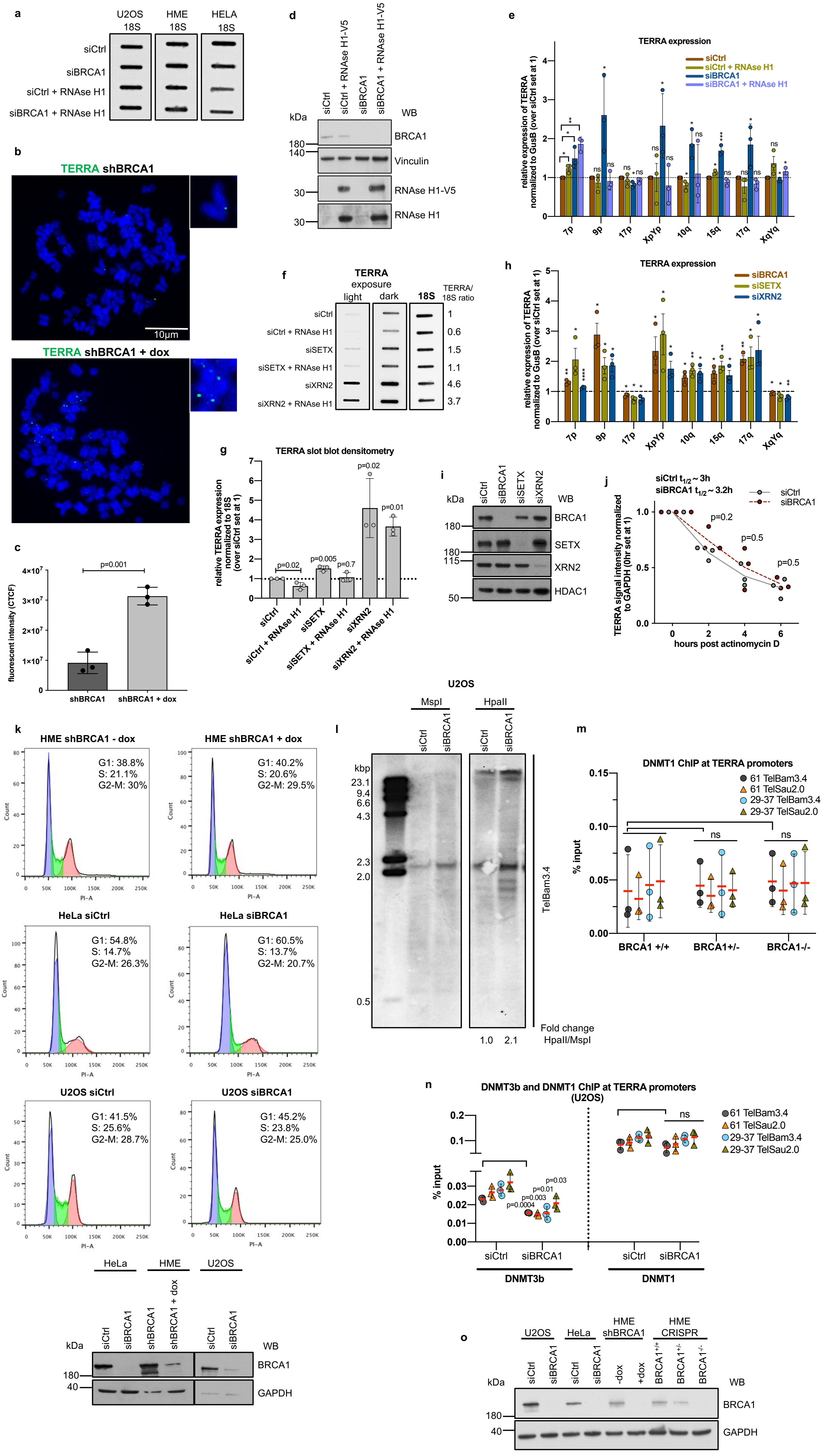
a
U2OS 18S
HME 18S
HELA 18S
siCtrl
siBRCA1
siCtrl + RNAse H1
siBRCA1 + RNAse H1
b
TERRA shBRCA1
10µm
TERRA shBRCA1 + dox
c
p=0.001
fluorescent intensity (CTCF)
shBRCA1
shBRCA1 + dox
d
siCtrl
siCtrl + RNAse H1-V5
siBRCA1
siBRCA1 + RNAse H1-V5
kDa
WB
BRCA1
Vinculin
RNAse H1-V5
RNAse H1
e
TERRA expression
siCtrl
siCtrl + RNAse H1
siBRCA1
siBRCA1 + RNAse H1
relative expression of TERRA normalized to GusB (over siCtrl set at 1)
7p
9p
17p
XpYp
10q
15q
17q
XqYq
f
TERRA exposure
light
dark
18S
TERRA/18S ratio
siCtrl 1
siCtrl + RNAse H1 0.6
siSETX 1.5
siSETX + RNAse H1 1.1
siXRN2 4.6
siXRN2 + RNAse H1 3.7
g
TERRA slot blot densitometry
relative TERRA expression normalized to 18S (over siCtrl set at 1)
p=0.02
p=0.005
p=0.7
p=0.02
p=0.01
siCtrl
siCtrl + RNAse H1
siSETX
siSETX + RNAse H1
siXRN2
siXRN2 + RNAse H1
h
TERRA expression
siBRCA1
siSETX
siXRN2
relative expression of TERRA normalized to GusB (over siCtrl set at 1)
i
kDa
siCtrl
siBRCA1
siSETX
siXRN2
WB
BRCA1
SETX
XRN2
HDAC1
j
siCtrl t1/2 ~ 3h
siBRCA1 t1/2 ~ 3.2h
siCtrl
siBRCA1
p=0.2
p=0.5
p=0.5
TERRA signal intensity normalized to GAPDH (0hr set at 1)
hours post actinomycin D
k
HME shBRCA1 - dox
G1: 38.8%
S: 21.1%
G2-M: 30%
HME shBRCA1 + dox
G1: 40.2%
S: 20.6%
G2-M: 29.5%
HeLa siCtrl
G1: 54.8%
S: 14.7%
G2-M: 26.3%
HeLa siBRCA1
G1: 60.5%
S: 13.7%
G2-M: 20.7%
U2OS siCtrl
G1: 41.5%
S: 25.6%
G2-M: 28.7%
U2OS siBRCA1
G1: 45.2%
S: 23.8%
G2-M: 25.0%
Count
PI-A
HeLa
HME
U2OS
siCtrl
siBRCA1
shBRCA1
shBRCA1 + dox
kDa
WB
BRCA1
GAPDH
l
U2OS
MspI
HpaII
siCtrl
siBRCA1
kbp
23.1
9.4
6.6
4.3
2.3
2.0
0.5
TelBam3.4
Fold change HpaII/MspI
1.0
2.1
m
DNMT1 ChIP at TERRA promoters
61 TelBam3.4
61 TelSau2.0
29-37 TelBam3.4
29-37 TelSau2.0
% input
ns
ns
BRCA1 +/+
BRCA1+/-
BRCA1-/-
n
DNMT3b and DNMT1 ChIP at TERRA promoters (U2OS)
% input
p=0.0004
p=0.003
p=0.01
p=0.03
ns
siCtrl
siBRCA1
DNMT3b
DNMT1
o
U2OS
HeLa
HME shBRCA1
HME CRISPR
siCtrl
siBRCA1
-dox
+dox
BRCA1+/+
BRCA1+/-
BRCA1-/-
kDa
WB
BRCA1
GAPDH

Supplementary Figure 4. BRCA1 absence leads to increased TERRA levels

a. Slot blot analysis of 18S signals used for normalization in **Fig. 3a.** n=3 independent experiments. **b.** Staining of TERRA (green) in metaphase spreads of control and BRCA1-depleted HME cells. **c.** Measurement of TERRA fluorescent intensity in **b**, n=3 independent experiments, ±SD (two-tailed Student's t-test). **d.** Immunoblot confirming the efficiency of BRCA1 depletion and the expression of V5-tagged RNAse H1-encoding vector. n=3 independent experiments. **e.** qRT-PCR analysis of TERRA levels from individual subtelomeres in mock-, or RNAse H1-treated control and BRCA1-depleted U2OS cells. The data were normalized to GusB and compared to siCtrl, set at 1, n=3 independent experiments, ±SD. p-values were computed using a two-tailed Student's t-test; $^{*}p < 0.05$, $^{**}p < 0.005$, $^{***}p < 0.0005$, ns – not significant. **f-g.** Slot blot and respective densitometry analyses of TERRA expression in mock-, or RNAse H1-treated siCtrl, siSETX, and siXRN2 samples from U2OS cells. Bars represent the average value of TERRA signals, normalized to 18S, n=3 independent experiments, ±SD. p-values were calculated using a two-tailed Student's t-test. **h.** qRT-PCR analysis of TERRA levels from different subtelomeres in siCtrl, siBRCA1, siSETX, and siXRN2 U2OS cells . The data were normalized to GusB and compared to siCtrl, set at 1, n=3 independent experiments, ±SD. p-values were computed using a two-tailed Student's t-test; $^{*}p < 0.05$, $^{**}p < 0.005$, $^{***}p < 0.0005$, ns – not significant. **i.** Immunoblot confirming depletion of BRCA1, SETX, and XRN2. n=3 independent experiments. **j.** Control and BRCA1-depleted HeLa cells were treated with 5 $\mu g/ml^{-1}$ actinomycin D for the indicated times. TERRA signal was normalized with GAPDH. Each dot represents a biological replicate (n=3), and the mean values are connected by a line. p-values were calculated using a two-tailed Student's t-test. **k.** Cell cycle distribution using FACS analysis and respective immunoblots of control and BRCA1-depleted HeLa, U2OS, and HME cells. n=3 independent experiments. **l.** TERRA CpG-island promoter methylation analysis of control and BRCA1-depleted U2OS cells. Genomic DNA was digested with MspI and HpaII restriction enzymes and hybridized using a DIG-labelled probe detecting TERRA promoter CpG-island repeats. n=3 independent experiments. **m-n.** ChIP analyses of promoter regions performed in HME (**m**) and control as well as BRCA1-depleted U2OS cells (**n**) using DNMT3b and/or DNMT1 antibodies, n=3 independent experiments, ±SD. p-values were calculated using a two-tailed Student's t-test, ns – not significant. **o.** Immunoblot confirming BRCA1 depletion in the indicated cell lines (**Fig. 3l**). n=3 independent experiments. Source data are provided as a Source data file.

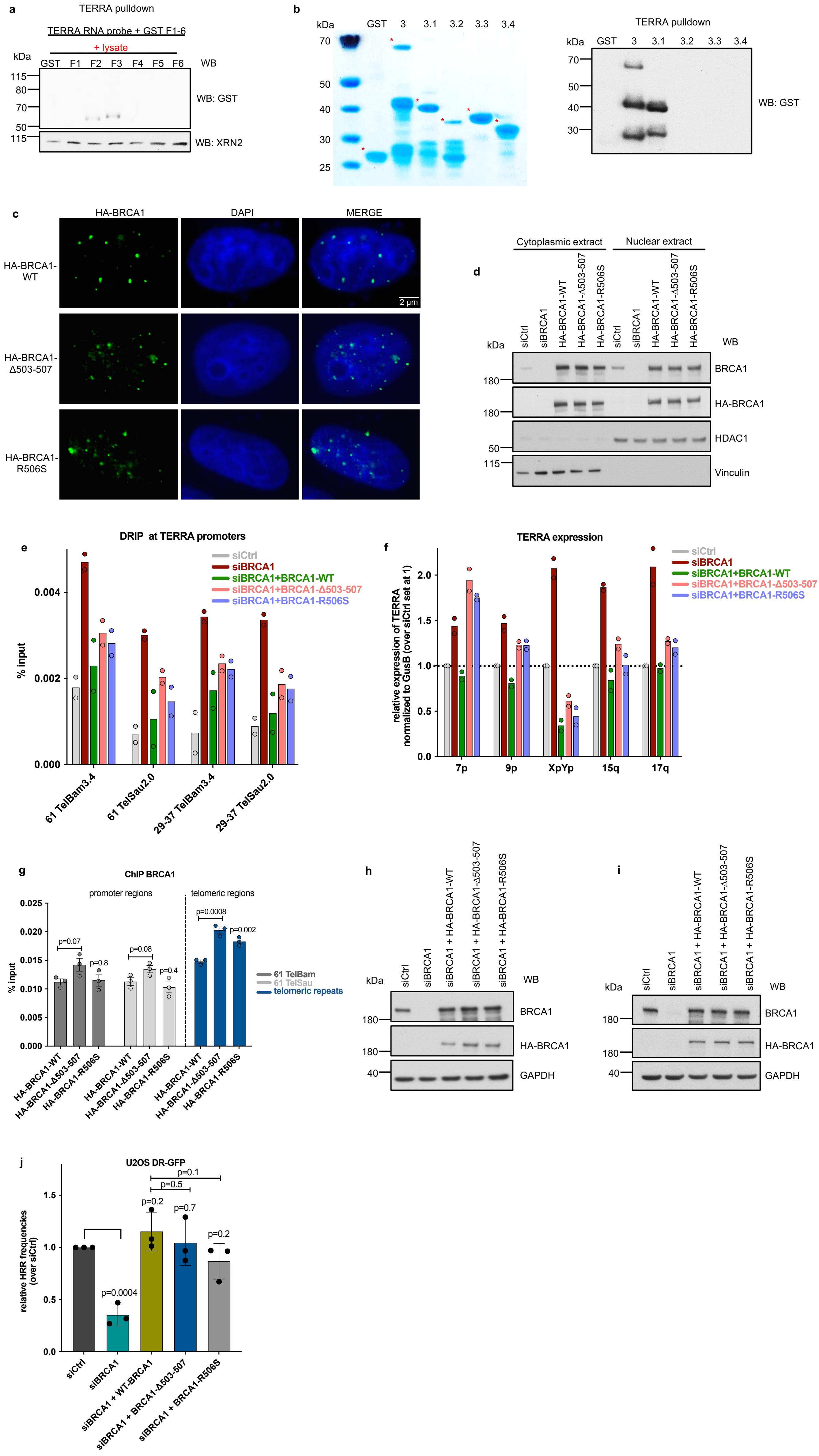
a
TERRA pulldown
TERRA RNA probe + GST F1-6
+ lysate
kDa
GST F1 F2 F3 F4 F5 F6
WB
115
80
70
50
WB: GST
115
WB: XRN2
b
kDa
GST 3 3.1 3.2 3.3 3.4
70
50
40
30
25
TERRA pulldown
kDa
GST 3 3.1 3.2 3.3 3.4
70
50
40
30
WB: GST
c
HA-BRCA1
DAPI
MERGE
HA-BRCA1-WT
2 μm
HA-BRCA1-Δ503-507
HA-BRCA1-R506S
d
Cytoplasmic extract
Nuclear extract
siCtrl
siBRCA1
HA-BRCA1-WT
HA-BRCA1-Δ503-507
HA-BRCA1-R506S
siCtrl
siBRCA1
HA-BRCA1-WT
HA-BRCA1-Δ503-507
HA-BRCA1-R506S
WB
kDa
180
BRCA1
180
HA-BRCA1
50
HDAC1
115
Vinculin
e
DRIP at TERRA promoters
siCtrl
siBRCA1
siBRCA1+BRCA1-WT
siBRCA1+BRCA1-Δ503-507
siBRCA1+BRCA1-R506S
% input
0.004
0.002
0.000
61 TelBam3.4
61 TelSau2.0
29-37 TelBam3.4
29-37 TelSau2.0
f
TERRA expression
siCtrl
siBRCA1
siBRCA1+BRCA1-WT
siBRCA1+BRCA1-Δ503-507
siBRCA1+BRCA1-R506S
relative expression of TERRA normalized to GusB (over siCtrl set at 1)
2.0
1.5
1.0
0.5
0.0
7p
9p
XpYp
15q
17q
g
ChIP BRCA1
promoter regions
telomeric regions
% input
0.025
0.020
0.015
0.010
0.005
0.000
p=0.07
p=0.8
p=0.08
p=0.4
p=0.0008
p=0.002
61 TelBam
61 TelSau
telomeric repeats
HA-BRCA1-WT
HA-BRCA1-Δ503-507
HA-BRCA1-R506S
HA-BRCA1-WT
HA-BRCA1-Δ503-507
HA-BRCA1-R506S
HA-BRCA1-WT
HA-BRCA1-Δ503-507
HA-BRCA1-R506S
h
kDa
siCtrl
siBRCA1
siBRCA1 + HA-BRCA1-WT
siBRCA1 + HA-BRCA1-Δ503-507
siBRCA1 + HA-BRCA1-R506S
WB
180
BRCA1
180
HA-BRCA1
40
GAPDH
i
kDa
siCtrl
siBRCA1
siBRCA1 + HA-BRCA1-WT
siBRCA1 + HA-BRCA1-Δ503-507
siBRCA1 + HA-BRCA1-R506S
WB
180
BRCA1
180
HA-BRCA1
40
GAPDH
j
U2OS DR-GFP
relative HRR frequencies (over siCtrl)
1.5
1.0
0.5
0.0
p=0.1
p=0.5
p=0.2
p=0.7
p=0.2
p=0.0004
siCtrl
siBRCA1
siBRCA1 + WT-BRCA1
siBRCA1 + BRCA1-Δ503-507
siBRCA1 + BRCA1-R506S

Supplementary Figure 5. TERRA interaction-associated BRCA1 mutants are nuclear and display normal, non-telomeric HRR properties

a. TERRA pulldown assays using BRCA1-GST fragments. A biotinylated TERRA probe was incubated with the GST-BRCA1 fusion proteins described in **Fig. 4a** in the presence of HeLa CF. The complexes were analyzed by immunoblot using anti-GST antibody. TERRA binding to XRN2 served as a positive control. n=3 independent experiments. **b.** TERRA pulldown assay using a series of truncated BRCA1-GST fragments. A biotinylated TERRA probe was incubated with the GST-BRCA1 fusion proteins only, and the pulldown was analyzed by immunoblotting with anti-GST antibody. Coomassie blue staining was used to examine the relative abundance of GST fusion proteins. n=3 independent experiments. **c-d.** Immunofluorescent staining and immunoblot of HA-tagged WT, ΔNLS1, and R506S BRCA1 mutants using anti-HA antibody to test nuclear localization of the aforementioned proteins. Expression of vinculin (cytoplasmic) and HDAC1 (nuclear) proteins was used to examine the efficiency of the relevant fractionation processes. n=3 independent experiments. **e.** DRIP analyses of promoter regions performed in WT and mutant BRCA1-expressing U2OS cells. Bars represent the average value of n=2 biological replicates. **f.** TERRA levels in WT and mutant BRCA1-expressing U2OS cells from different subtelomeres, normalized to GusB and compared to siCtrl, set at 1. Bars represent the average value of n=2 biological replicates. **g.** Association of HA-tagged WT and mutant BRCA1 proteins with TERRA promoters and telomere DNA was assessed by ChIP using anti-BRCA1 antibody. Bars represent the average value of n=3 biological replicates, ±SD. p-values were computed using a two-tailed Student's t-test. **h-i.** Immunoblots of U2OS cells used in **e-g** and **Fig**. **5h**, confirming the absence of endogenous BRCA1 and efficient expression of HA-tagged WT and mutant BRCA1. n=3 independent experiments. **j.** HRR assay in U2OS cells expressing HA-BRCA1-WT, HA-BRCA1-ΔNLS1, and HA-BRCA1-R506S. Along with transfection of the I-SceI vector, transfection of the dsRed vector was used to estimate transfection efficiency. The HRR frequencies of the control sample were set at 1. n=10 000 cells were scored per experiment, and graphs represent the mean GFP/dsRED signal from three independent experiments, ±SD. p-values were computed using a two-tailed Student's t-test. Source data are provided as a Source data file.

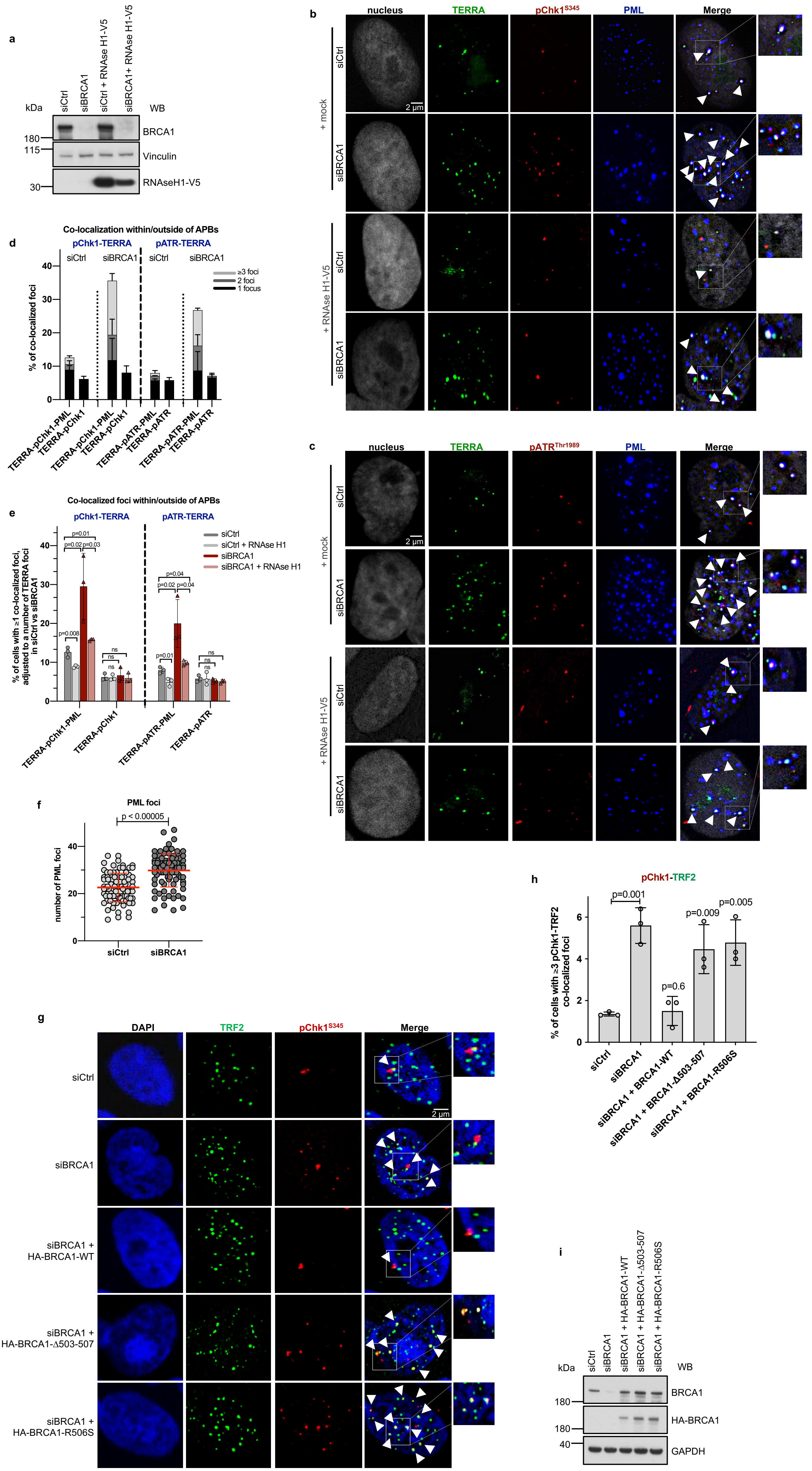

a
kDa
siCtrl
siBRCA1
siCtrl + RNAse H1-V5
siBRCA1+ RNAse H1-V5
WB
BRCA1
180
115
Vinculin
30
RNAseH1-V5
b
nucleus
TERRA
pChk1S345
PML
Merge
+ mock
siCtrl
siBRCA1
+ RNAse H1-V5
siCtrl
siBRCA1
2 µm
c
nucleus
TERRA
pATRThr1989
PML
Merge
+ mock
siCtrl
siBRCA1
+ RNAse H1-V5
siCtrl
siBRCA1
2 µm
d
Co-localization within/outside of APBs
pChk1-TERRA
pATR-TERRA
siCtrl
siBRCA1
siCtrl
siBRCA1
≥3 foci
2 foci
1 focus
% of co-localized foci
40
30
20
10
0
TERRA-pChk1-PML
TERRA-pChk1
TERRA-pChk1-PML
TERRA-pChk1
TERRA-pATR-PML
TERRA-pATR
TERRA-pATR-PML
TERRA-pATR
e
Co-localized foci within/outside of APBs
pChk1-TERRA
pATR-TERRA
siCtrl
siCtrl + RNAse H1
siBRCA1
siBRCA1 + RNAse H1
% of cells with ≥1 co-localized foci, adjusted to a number of TERRA foci in siCtrl vs siBRCA1
p=0.01
p=0.02
p=0.03
p=0.008
ns
ns
ns
p=0.04
p=0.02
p=0.04
p=0.01
ns
ns
ns
40
30
20
10
0
TERRA-pChk1-PML
TERRA-pChk1
TERRA-pATR-PML
TERRA-pATR
f
PML foci
p < 0.00005
number of PML foci
40
20
0
siCtrl
siBRCA1
g
DAPI
TRF2
pChk1S345
Merge
siCtrl
siBRCA1
siBRCA1 + HA-BRCA1-WT
siBRCA1 + HA-BRCA1-Δ503-507
siBRCA1 + HA-BRCA1-R506S
2 µm
h
pChk1-TRF2
% of cells with ≥3 pChk1-TRF2 co-localized foci
p=0.001
p=0.6
p=0.009
p=0.005
6
4
2
0
siCtrl
siBRCA1
siBRCA1 + BRCA1-WT
siBRCA1 + BRCA1-Δ503-507
siBRCA1 + BRCA1-R506S
i
kDa
siCtrl
siBRCA1
siBRCA1 + HA-BRCA1-WT
siBRCA1 + HA-BRCA1-Δ503-507
siBRCA1 + HA-BRCA1-R506S
WB
BRCA1
180
HA-BRCA1
180
40
GAPDH

Supplementary Figure 6. BRCA1 suppresses replication stress at telomeres
a. An immunoblot that confirms the efficiencies of BRCA1 depletion and expression of V5-tagged RNAse H1-encoding vector in U2OS cells. n=3 independent experiments. **b-c.** Representative images of TERRA RNA-FISH combined with anti-PML and anti- pChk1^{S345} (**b**) or anti- pATR^{Thr1989} (**c**) immunostaining performed in mock-, or RNAse H1-treated siCtrl and BRCA1-depleted U2OS cells. Triple co-localization events are depicted with arrowheads and dual co-localizations with asterisks, respectively. n=3 independent experiments. **d.** Mean percentage of cells with one, two, or ≥3 TERRA-PML-pChk1^{S345}/pATR ^{Thr1989} co-localized foci in control and BRCA1-depleted U2OS cells from three independent experiments. At least n=70 cells scored/experiment, ±SD. **e.** Mean percentage of ≥1 TERRA-PML-pChk1^{S345}/pATR ^{Thr1989} co-localization events per nucleus, adjusted to a number of TERRA foci in mock-, or RNAse H1-treated control and BRCA1-depleted U2OS cells from three independent experiments. At least n=70 cells scored/experiment, ±SD. p-values were obtained using a two-tailed Student's t-test, ns – not significant. **f.** Quantitation of PML foci in control and BRCA1-depleted U2OS cells from three independent experiments. At least n=30 cells scored/experiment, ±SD. p-value was assessed using a two-tailed Student's t-test. **g.** Representative images of combined immunofluorescence using anti-TRF2 and anti- pChk1^{S345} immunostaining performed in U2OS cells, depleted of endogenous BRCA1 and transfected with WT and mutant BRCA1 vectors. Co-localization events are depicted with arrowheads. n=3 independent experiments. **h.** Mean percentage of cells with ≥3 TRF2-pChk1^{S345} co-localized foci depicted in **g** from three independent experiments. At least n=80 cells scored/experiment, ±SD. p-values were computed using a two-tailed Student's t-test. **i.** Immunoblot of U2OS cells used in **g-h** and **Fig. 5d-e**, confirming the absence of endogenous BRCA1 and efficient expression of HA-tagged WT and mutant BRCA1. n=3 independent experiments. Source data are provided as a Source data file.

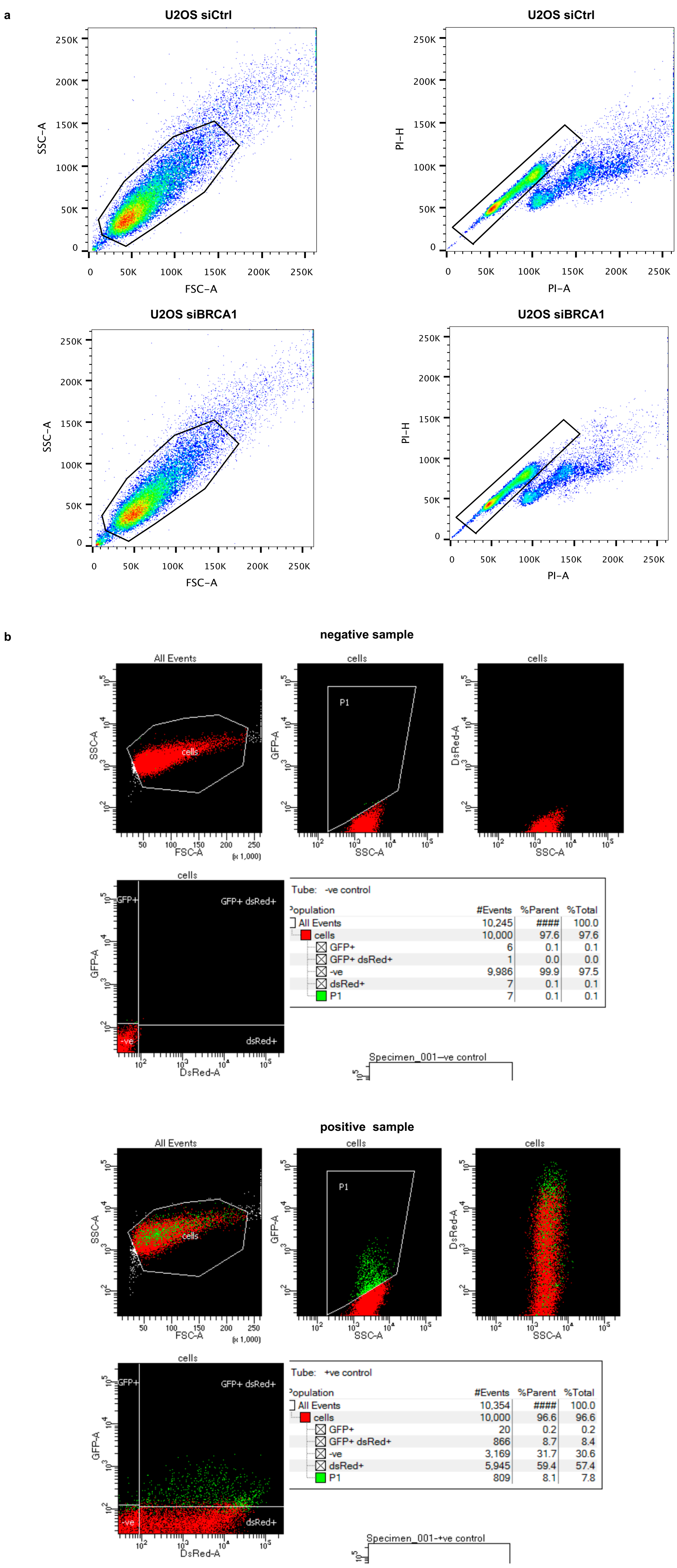
a
U2OS siCtrl
SSC-A
FSC-A
PI-H
PI-A
U2OS siBRCA1
b
negative sample
All Events
cells
P1
SSC-A
FSC-A
GFP-A
DsRed-A
GFP+
GFP+ dsRed+
-ve
dsRed+
Tube: -ve control
Population | #Events | %Parent | %Total
All Events | 10,245 | #### | 100.0
cells | 10,000 | 97.6 | 97.6
GFP+ | 6 | 0.1 | 0.1
GFP+ dsRed+ | 1 | 0.0 | 0.0
-ve | 9,986 | 99.9 | 97.5
dsRed+ | 7 | 0.1 | 0.1
P1 | 7 | 0.1 | 0.1
Specimen_001-ve control
positive sample
Tube: +ve control
Population | #Events | %Parent | %Total
All Events | 10,354 | #### | 100.0
cells | 10,000 | 96.6 | 96.6
GFP+ | 20 | 0.2 | 0.2
GFP+ dsRed+ | 866 | 8.7 | 8.4
-ve | 3,169 | 31.7 | 30.6
dsRed+ | 5,945 | 59.4 | 57.4
P1 | 809 | 8.1 | 7.8
Specimen_001-+ve control

Supplementary Figure 7. Gating strategy for FACS analyses
Representative gating strategies for Supplementary Fig. 4k (**a**) and Supplementary Fig. 5j (**b**). Source data are provided as a Source data file.

Supplementary Tables

Supplementary Table 1. Lists of proteins identified in BRCA1 and TRF2 pulldown assays.

Proteins detected by proteomics analysis of immunoprecipitated BRCA1	Proteins detected by proteomics analysis of immunoprecipitated TRF2	Proteins commonly detected in BRCA1 and TRF2 immunoprecipitates by nanoLC-MS
**Gene|Gene Groups**	**Gene|Gene Groups**	**Gene Name**
PKM	C10orf12	DDX27
DOCK7	RANBP2	SF3B3
DDX27	ZMYM2	HNRNPUL1
SF3B3	**TERF2**	FTSJ3
HNRNPUL1	SUZ12	U2SURP
FTSJ3	TERF2IP	NKRF
U2SURP	EHMT1	DDX54
EEF2	EHMT2	CHERP
NKRF	MKI67	XRN2
FAM83H	ZNF518A	RPL5
ADARB1	EZH2	UBTF
APOB	FLNB	TBL3
DDX54	RCC1	SF3A1
CHERP	LCOR	RPS24
HSP90AB1	H2AFY	NIFK
XRN2	EED	MRTO4
RPL5	SNRNP70	RPL36
WDR36	PHF14	TRA2A
UBTF	SF3B3	WDR75
TBL3	DDX27	TEX10
SF3A1	PHF1	SF3B2
**BRCA1**	PRPF6	KIAA0020
RPS24	SEPT9	TRIM28
NIFK	ACD	SLTM
STAU1	TCF20	WDR12
MRTO4	TRAF4	RBM12B
RPL36	PKP3	RBM14
CCT8	RUVBL1	PKP3
F2	FTSJ3	BAZ1B
TRA2A	SF3A1	NOP16
PRMT1	C1QBP	RPL23
WDR75	VASP	CHTOP
WDR74	DDX23	UTP20
TGM2	CCDC86	SCAF1

TEX10	TINF2	FUS
SUGP2	HSPA9	RCL1
TCP1	NUP133	RRP7A
SF3B2	SCAF1	HIST1H1A
KIAA0020	CDC40	RPL15
TRIM28	TRA2A	SNRPD3
SLTM	NUP88	AATF
NOP14	SF3B2	UTP14A; UTP14C
LRP1	SNRPD3	H2AFY
ZCCHC8	WIZ	CCDC86
AQR	RPL5	DCAF13
WDR12	MRTO4	RCC2
RBM12B	RCC2	RPL30
RBM14	DDX47	SQRDL
PKP3	SMC3	PES1
BAZ1B	RPL23A	PRPF6
PSME1	CPSF6	DNTTIP2
TRIM29	WDR75	PPAN; PPAN-P2RY11
NOP16	DNTTIP2	LOC101929876; RPS26
RPL23	BUB3	CBX5
CHTOP	CBX1	HNRNPAB
CARM1	H1FX	RPS4X
SUPT5H	KIAA0020	RTCB
AKAP8	DYNC1H1	LAS1L
RRP1B	DDX24	LYAR
BMS1	RPL15	RRP9
CCT2	SNRNP40	RRP12
CCT4	TPR	DDX31
CCT5	CLTB	RPS7
ATAD2	SMC1A	CRNKL1
UTP20	NUP155	HP1BP3
SCAF1	POT1	SNRPE
F5	ALDH18A1	H2AFY; H2AFY2
UTP14A	SNRPE	TOP1
FUS	PSPC1	MYBBP1A
FUBP3	MYBBP1A	FLNB
DDX51	BANF1	NUP133
DDX56	CPSF1	SMC3
SRRT	UBTF	CDC40
UTP15	NOLC1	IMP3
RCL1	BAZ1B	SFN
CEBPZ	TBL3	RPLP0

RRP7A	CABIN1	C1QBP
HIST1H1A	RUVBL2	RPS3A
RPL15	DLAT	DYNC1H1
SNRPD3	EMD	EMD
AATF	TEX10	SENP3
PGK1	HNRNPAB	SNRNP40
UPF1	U2SURP	EEF1G
GNB2L1	RPRD1B	NHP2
UTP14A; UTP14C	EEF1B2	SMC1A
MYO1D	CRNKL1	NUP155
SURF6	TRIM28	SMCHD1
H2AFY	LAS1L	HNRNPD
CCDC86	CHERP	SNRPB; SNRPN
ITPR3	SNRPD2	SNRPG
RBM22	SNRPD1	
MAK16	SLTM	
DCAF13	SEPT7	
RCC2	XRCC6	
RPL30	H2AFY2	
SQRDL	RPS7	
WDR3	RPS24	
HSP90AA1; HSP90AB1	DDX31	
CCT3	THOC6	
KRT18	THOC5	
ZNF638	NKRF	
EIF2S3	TERF1	
HEATR1	XRN2	
KPNB1	NUP205	
PES1	SMARCC1; SMARCC2	
PRPF6	ZNF281	
DNTTIP2	KDM1A	
NOL10	SMCHD1	
PPAN; PPAN-P2RY11	NIFK	
CNN3	HMGA1	
KHDRBS1	HP1BP3	
DDX52	DCAF13	
LOC101929876; RPS26	RPL30	
SUPT6H	SNRPB; SNRPN	
RPL7L1	RPL36	
CBX5	MFAP4	
MLF2	RGPD1	
NOC2L	H2AFY; H2AFY2	

RBM4; RBM4B	SQRDL	
HNRNPAB	CTCF	
UTP18	PDS5A	
VTN	SENP3	
ENO1	RBM12B	
RPF1	ARPC4; ARPC4-TTLL3	
RPS4X	TOP1	
RTCB	PRDM2	
SF3B4	RRP12	
OTUB1	EZH1; EZH2	
NKTR	RBM15	
LAS1L	RBM14	
PGK1; PGK2	CPSF2	
HBA1; HBA2	RANBP2	
MPHOSPH10	FIP1L1	
LYAR	RPL11	
RRP9	PES1	
UBA1	REXO4	
CTNND1	PPAN; PPAN-P2RY11	
RPSA	CENPV	
RRP12	UTP14A; UTP14C	
DDX31	RGPD3	
RPS7	SFN	
CIRH1A	FUS	
DOCK6; DOCK7	FUBP1	
FAM120A	LAMB3	
CRNKL1	KHSRP	
HSP90AA1	DDX54	
HP1BP3	SF3A3	
RPS18	LOC101929876; RPS26	
PLOD1	ALYREF	
SNRPE	RPLP0	
U2AF2	CBX5	
NCBP1	RRP8	
HSPA4	RCL1	
H2AFY; H2AFY2	RPS4X	
BYSL	RTCB	
PCBP1	RPS3A	
ABLIM1	CALD1	
CCT7	ZFR	
TOP1	RRP7A	
UTP23	NUP214	

MYBBP1A	CCDC71	
FLNB	LYAR	
NUP133	RRP9	
ITCH	NOP10	
SMC3	NOP16	
RBM17	XRCC5	
SARS	AATF	
CDC40	SEC13	
DDX10	PHF5A	
ITGB1	UTP11L	
ITGB4	MORF4L1	
MYO6	RPS28	
RBM14-RBM4; RBM4	WDR12	
PKP1	RPS4X; RPS4Y1	
TLN1	IMP3	
EIF3B	DCLRE1B	
STRAP	RPL23	
NOL11	FAU	
IMP3	LLPH	
CDKN2A	UTP20	
CENPC	NGDN	
PAFAH1B1	MRGBP	
SFN	S100A16	
C14orf169	LOC102724594; U2AF1	
TKT	HNRNPD	
EIF4E	CHTOP	
RSL24D1	EEF1G	
CLK1	NHP2	
CLK3	PDHX	
NUDCD1	TOP2B	
RRP1	ZNF518B	
RPLP2	HIST1H1A	
RPLP0	HNRNPUL1	
ZBTB11	MTF2	
C1QBP	SFN	
ZNF800	SNRPA	
RPS3A	SNRPF	
XAB2	SNRPG	
SF3B5	RPL38	
PSME2	PRPF38A	
DYNC1H1	RPL31	
TARS		

RBM42		
SPEN		
EMD		
SEPT2		
RBM34		
SENP3		
TUBB1		
KNOP1		
GDI2		
BCAS2		
SRM		
USP7		
CSNK1A1		
FARSB		
FARSA		
PPP2R1A		
MDH1		
NOC4L		
ALDOA		
ALDOC		
SNRNP40		
RRP15		
S100A14		
EEF1G		
NHP2		
CHD2		
SMC1A		
ARPC3		
ARPC2		
POLR2A		
NUP155		
ACTR3		
SMCHD1		
NOM1		
ATP5B		
HNRNPD		
KIF20A		
UTP6		
FAM83A		
LDHB		
SNRPB; SNRPN		
SNRPG		

Supplementary Table 2. List of antibodies used in this study.

Antibodies	Source	Dilution
SETX (WB)	Bethyl Labs, Cat#A301-104A	1:1000
XRN2 (H-3) (IP)	Santa Cruz, Cat#sc365258	5 µg/200µg extract
XRN2 (WB)	Bethyl Labs, Cat#A301-103A	1:1000
GAPDH (WB)	Santa Cruz, Cat#sc47724	1:5000
TRF1-78 (IP, WB)	Santa Cruz, Cat#sc56807	1:100 (WB), 5 µg/200µg extract (IP)
Cyclin A (WB)	Santa Cruz, Cat#sc271682	1:2000
Geminin (WB)	Santa Cruz, Cat#FL-209	1:2000
TRF2 (WB)	Bethyl Labs, Cat#A300-796A	1:1000
TRF2 (ChIP grade) (IP)	Abcam, Cat#ab13579	2 µg/200µg extract
RAP1 (IP, WB)	Santa Cruz, Cat#sc53434	1:100 (WB), 3 µg/200µg extract (IP)
GST (WB)	Bethyl Labs, Cat#A190-122A	1:2000
rabbit IgG (IP)	Santa Cruz, Cat#sc2027	3 µg/200µg extract
mouse IgG (IP)	Millipore, Cat#12-371	3 µg/200µg extract
Cyclin E (WB)	BD Pharmingen, Cat#BDB551159	1:2000
TIN2 (IP)	Abcam, Cat#ab197894	2 µg/200µg extract
TIN2 (WB)	Origene, Cat#CF809776	1:1000
TPP1 (IP)	Abcam, Cat#ab112050	2 µg/200µg extract
TPP (WB)	Bethyl, Cat#A303-069A	1:1000
53BP1 (WB)	Novus Biologicals, Cat#NB100-304	1:5000
POT1 (WB)	Biovision, Cat#A1916	1:500
POT1 (IP)	Proteintech, Cat#10581-1-AP	3 µg/200µg extract
anti-HA.11 (WB)	Biolegend, Cat#16B12	1:2000
p27 (WB)	BD Biosciences, Cat#BD610241	1:1000
pH3(Ser10) (WB)	Millipore, Cat#06-570	1:5000
BRCA1 SD118 (IP, WB)	Millipore, Cat#OP107	1:500 (WB), 3 µg/200µg extract (IP)
BRCA2 (WB)	Bethyl Labs, Cat#A300-005A	1:1000
Vinculin (G-11) (WB)	Santa Cruz, Cat#sc-55465	1:5000
BARD1 (WB)	Bethyl Labs, Cat#A300-263A	1:1000
MDC1 (WB)	Bethyl Labs, Cat#A300-052A	1:1000
RPA34 (WB)	Millipore, Cat#NA19L	1:1000
GFP (WB)	Novus Biologicals, Cat#NB600308	1:2000
RNAse H1 (WB)	Genetex, Cat#GTX117624	1:500
V5-tag (D3H8Q) (WB)	Cell Signaling, Cat#13202	1:5000
HDAC1 (WB)	Cell Signaling, Cat#34589	1:5000
BRCA1 (IF)	Millipore, Cat#07-434	1:800
pChk1^{S345} (IF)	Life Technologies, Cat#MA515145	1:500
pATR^{1989} (IF)	Genetex, Cat#GTX128145	1:1000
TRF2 (IF)	Abcam, Cat#ab13579	1:1000
PML (IF)	Abcam, Cat#ab96051	1:500
HA (IF)	Biolegend, Cat#16B12	1:1000
γH2AX (IF)	Millipore, Cat#05-636	1:300
ssDNA (slot blot)	Millipore, Cat#MAB3034	1:250
S9.6 (slot blot)	Millipore, Cat#MABE1095	1:500
S9.6 (DRIP)	DFCI Monoclonal antibody Core	2.5µg/15µg sheared DNA
RNA Polymerase II (ChIP)	Biolegend, Cat#8WG16	4µg/20µg sheared DNA
RNA-PolII-(S2) (ChIP)	Bethyl, Cat#A300-654A	1µg/20µg sheared DNA
RNA-PolII-(S5) (ChIP)	Bethyl, Cat#A304-408A	1µg/20µg sheared DNA
BRCA1 (ChIP)	Bethyl, Cat#A300-000A	1µg/20µg sheared DNA
DNMT3b (ChIP)	Novus Biologicals, Cat#NB300516	2µg/20µg sheared DNA

DNMT1 (ChIP)	Novus Biologicals, Cat#NB100-56519	1μg/20μg sheared DNA
H3K27me3 (ChIP)	Abcam, Cat#ab6002	2μg/20μg sheared DNA
H3K9me3 (ChIP)	Abcam, Cat#8898	2μg/20μg sheared DNA
H3K4me3 (ChIP)	Abcam, Cat#ab8580	2μg/20μg sheared DNA
H4K16ac (ChIP)	Millipore, Cat#07329MI	5μg/20μg sheared DNA
H4 (ChIP)	Millipore, Cat#04858MI	5μg/20μg sheared DNA
H3 (ChIP)	Abcam, Cat#ab1791	2μg/20μg sheared DNA

Supplementary Table 3. List of primers used in this study.

Primers	Source
TERRA specific RT: 5’-CCCTAACCCTAACCCTAACCCTAACCCTAA-3’	[1]
GusB specific-RT: 5’-AATACAGATAGGCAGGGCGTTCG’-3	[1]
7p Frwd: 5’-CAATCTCGGCTCACCACAATC-3’	[2]
7p Rev: 5’-GGAGGCTGAGGCAGGAGAA-3’	[2]
9p Frwd: 5’-GAGATTCTCCCAAGGCAAGG-3’	[1]
9p Rev: 5’-ACATGAGGAATGTGGGTGTTAT-3’	[1]
17p Frwd: 5’-CTTATCCACTTCTGTCCCAAGG-3’	[1]
17p Rev: 5’-CCCAAAGTACACAAAGCAATCC-3’	[1]
XpYp Frwd: 5’-AAGAACGAAGCTTCCACAGTAT-3’	[1]
XpYp Rev: 5’-GGTGGGAGCAGATTAGAGAATAAA-3’	[1]
10q Frwd: 5’-ATGCACACATGACACCCTAAA-3’	[1]
10q Rev: 5’-TACCCGAACCTGAACCCTAA-3’	[1]
15q Frwd: 5’-CAGCGAGATTCTCCCAAGCTAAG-3’	[3]
15q Rev: 5’-AACCCTAACCACATGAGCAACG-3’	[3]
17q Frwd: 5’-GTCCATGCATTCTCCATTGATAAG-3’	[2]
17q Rev: 5’-AGCTACCTCTCTCAACACCAAGAAG-3’	[2]
20q Frwd: 5’-ACATGGGCGATACTCAGG-3’	[4]
20q Rev: 5’-CCCACTACTGTGCCTCAA-3’	[4]
XqYq Frwd: 5’-GAAAGCAAAAGCCCCTCTGA-3’	[2]
XqYq Rev: 5’-CCCCTTGCCTTGGGAGAA-3’	[2]
TelBam3.4 61 Frwd: 5’-CGTGCCACCACTATACAGTAA-3’	This study
TelBam3.4 61 Rev: 5’-AACTGCAGGGCTCTCTTG-3’	This study
TelBam3.4 29-37 Frwd: 5’-CAGAGTTCTCCTCAGGTCAGA-3’	This study
TelBam3.4 29-37 Rev: 5’-GGAATGCTGCTCCACCTTTA-3’	This study
TelSau2.0 61 Frwd: 5’-CCACTATACTGCGAGCAAGAG-3’	This study
TelSau2.0 61 Rev: 5’-TACTGGCGACTAGGACAACT-3’	This study
TelSau2.0 29-37 Frwd: 5’-GCGGAGTTGCGTTCTCTTTA-3’	This study

TelSau2.0 29-37 Rev: 5'-CTCCGCCTTCGCAATACC-3'	This study
Telomeric repeats Frwd: 5'-<br>GGTTTTTGAGGGTGAGGGTGAGGGTGAGGGTGAGGGT-3'	[5]
Telomeric repeats Rev: 5'-<br>TCCCGACTATCCCTATCCCTATCCCTATCCCTATCCCTA-3'	[5]
TelBam3.4 DIG PCR Frwd: 5'-CACCACTCTAAGCAAGAGAGC-3'	This study
TelBam3.4 DIG PCR Rev: 5'- TTCGCAGTACCACCGAAATC-3'	This study

Supplementary Methods

Cell lines
Genetically modified U2OS DR-GFP cells[6] were grown in DMEM supplemented with 10% FBS and 50 µg/ml penicillin-streptomycin. All cells were cultivated in a humidified incubator at 37^0C in a 5% CO2-containing atmosphere.

siRNAs
All siRNAs were synthesized in Dharmacon Inc: siSETX 5'-GCCAGAUCGUAUACAAUUAUU-3', siTRF2 5'-GAACAAGCGCAUGACAAUAUU-3', siXRN2_10 5'- GGAAAGUUGUGCAGUCGUA-3', siXRN2_11 5'-UCGUUAGAGAUUAGGGAAA-3' (siXRN2 contains 50% of siXRN2_10 and 50% of siXRN2_11). siRNA oligos were introduced at a final concentration of 20nM by reverse transfection using RNAiMax (Invitrogen) according to the manufacturer's instructions. Cells were then incubated for 72h following transfection.

Plasmid vectors
Transfection of all vectors was performed using Lipofectamine 2000 (Invitrogen) according to the manufacturer's instructions. The dsRed (11151) vector was purchased from Addgene. GST-F3-BRCA1 fragments (3.1-3.4) have been described[7].

Cellular protein fractionation
Protein fractionation for **Supplementary Fig. 5f** was performed using a Subcellular protein fractionation kit (Thermo Scientific) according to manufacturer's instructions.

Cell treatments
To measure the half-life of TERRA, HeLa cells were treated with 5 µg/ml actinomycin D (Sigma) and collected every 2 hours.

R-loop detection using slot blot
For the detection of R-loops in **Supplementary Fig. 1j**, genomic DNA was extracted using a Genomic DNA extraction kit (Purelink), and 800 ng were loaded onto 0.2µm nitrocellulose membranes. Membranes were baked for 2 hours at 80°C, blocked for 1 hour in Odyssey Blocking buffer (Fisher Scientific), and incubated with S9.6 antibody overnight at 4^0C. Following washes in 1xTBST, membranes were incubated with secondary antibody IRDye 680LT (1:10.000, Li-Cor 926-6802) and washed again. Membranes were analyzed with a Li-Cor Odyssey CLx scanning apparatus using ImageStudioLite software. To probe for the loading control, membranes were denatured (0.4N NaOH, 0.5M NaCl) for 10 min, neutralized (1.5M NaCl, 0.5M Tris-HCl) for 5 minutes, and blocked for 1 hour. ssDNA antibody and secondary antibody IRDye 800CW (1:10.000, Li-Cor 926-32210) were used for normalization purposes.

RNA FISH staining in metaphase spreads
Cells were treated with colcemid (0.4µg/mL) for 4-6 hrs after which cells were harvested and incubated in 0.075 M KCl at 37°C for 20 min. Chromosomes were fixed in cold methanol/acetic acid (3:1), spread on glass slides, and air-dried overnight. The slides were re-hydrated and fixed with 4% PFA-PBS. Slides were then digested with pepsin (1 mg/ml) for 10 min at 37°C following fixation with 4% PFA-PBS. TERRA detection was performed using Tel-C-FAM probe (PNA Bio) without denaturation step (hybridization at RT for 4 hours).

Images were captured with Yokogawa spinning disk confocal on a Nikon Eclipse-TI inverted microscope and processed with ImageJ software.

HRR (Homologus Recombination Repair) Assay

U2OS cells[6] were transfected with HA-tagged BRCA1 constructs. The next day, cells were transfected with siCtrl and siRNA against BRCA1 3'UTR. The following day, cells were transfected with the I-SceI-encoding vector and incubated for 48 hours. In parallel, a vector expressing DsRed was transfected to determine transfection efficiency. Cells were then subjected to FACS analysis, and 10 000 cells were scored per experiment. Gating strategy is provided in Supplementary Fig. 7.

Cell cycle Analysis

Cell cycle distribution was analyzed based on DNA staining using Propidium Iodide (PI) (eBioscience) according to the manufacturer's protocol. Cells were washed with cold PBS, and fixed with cold 70% ethanol overnight. The PI staining was carried out by resuspending the cells in 500 μl PI/Triton X-100 solution (0.1% Triton X-100 in PBS, 0.2 mg/ml RNAse A, and PI 1 : 200) for 30 min at room temperature. Cell cycle data were acquired using a BD LSR Fortessa and the BD FACSDiva 8.0.1 software (BD Biosciences) and processed using FlowJo X.

References

1. Feretzaki, M. & Lingner, J. A practical qPCR approach to detect TERRA, the elusive telomeric repeat-containing RNA. *Methods* **114**, 39-45 (2017).
2. Deng, Z. et al. A role for CTCF and cohesin in subtelomere chromatin organization, TERRA transcription, and telomere end protection. *EMBO J* **31**, 4165-78 (2012).
3. Porro, A., Feuerhahn, S., Reichenbach, P. & Lingner, J. Molecular dissection of telomeric repeat-containing RNA biogenesis unveils the presence of distinct and multiple regulatory pathways. *Mol Cell Biol* **30**, 4808-17 (2010).
4. Montero, J.J., Lopez de Silanes, I., Grana, O. & Blasco, M.A. Telomeric RNAs are essential to maintain telomeres. *Nat Commun* **7**, 12534 (2016).
5. Cawthon, R.M. Telomere measurement by quantitative PCR. *Nucleic Acids Res* **30**, e47 (2002).
6. Rouet, P., Smih, F. & Jasin, M. Introduction of double-strand breaks into the genome of mouse cells by expression of a rare-cutting endonuclease. *Mol Cell Biol* **14**, 8096-106 (1994).
7. Hu, Y. et al. PARP1-driven poly-ADP-ribosylation regulates BRCA1 function in homologous recombination-mediated DNA repair. *Cancer Discov* **4**, 1430-47 (2014).
