# Supplementary material for: BRCA1 binds TERRA RNA and suppresses R-Loop-based telomeric DNA damage
Source: Nat Commun. 2021 Jun 10;12:3542. doi: 10.1038/s41467-021-23716-6 (PMC8192922; doi:10.1038/s41467-021-23716-6)
Supplement: Supplementary file 3 — Reporting Summary [file 41467_2021_23716_MOESM3_ESM.pdf]

nature research

Corresponding author(s): Jekaterina Vohhodina
David Livingston

Last updated by author(s): 2021/04/26

# Reporting Summary

Nature Research wishes to improve the reproducibility of the work that we publish. This form provides structure for consistency and transparency in reporting. For further information on Nature Research policies, see our Editorial Policies and the Editorial Policy Checklist.

## Statistics

For all statistical analyses, confirm that the following items are present in the figure legend, table legend, main text, or Methods section.

| n/a | Confirmed | |
|---|---|---|
| ☐ | ☒ | The exact sample size (*n*) for each experimental group/condition, given as a discrete number and unit of measurement |
| ☐ | ☒ | A statement on whether measurements were taken from distinct samples or whether the same sample was measured repeatedly |
| ☐ | ☒ | The statistical test(s) used AND whether they are one- or two-sided<br>*Only common tests should be described solely by name; describe more complex techniques in the Methods section.* |
| ☒ | ☐ | A description of all covariates tested |
| ☒ | ☐ | A description of any assumptions or corrections, such as tests of normality and adjustment for multiple comparisons |
| ☐ | ☒ | A full description of the statistical parameters including central tendency (e.g. means) or other basic estimates (e.g. regression coefficient) AND variation (e.g. standard deviation) or associated estimates of uncertainty (e.g. confidence intervals) |
| ☐ | ☒ | For null hypothesis testing, the test statistic (e.g. *F*, *t*, *r*) with confidence intervals, effect sizes, degrees of freedom and *P* value noted<br>*Give P values as exact values whenever suitable.* |
| ☒ | ☐ | For Bayesian analysis, information on the choice of priors and Markov chain Monte Carlo settings |
| ☒ | ☐ | For hierarchical and complex designs, identification of the appropriate level for tests and full reporting of outcomes |
| ☒ | ☐ | Estimates of effect sizes (e.g. Cohen's *d*, Pearson's *r*), indicating how they were calculated |

*Our web collection on statistics for biologists contains articles on many of the points above.*

## Software and code

Policy information about availability of computer code

| | |
|---|---|
| Data collection | Confocal images were captured with a Yokogawa spinning disk confocal on a Nikon Eclipse-TI inverted microscope and processed with ImageJ software.<br>Immunoblot membranes (R-loop detection using S9.6 antibody) were analyzed with a Li-Cor Odyssey CLx scanning apparatus using ImageStudioLite software.<br>qRT–PCR was performed in QuantStudio 6 Flex System (Applied Biosystems) using Applied Biosystems QuantStudio software. |
| Data analysis | Prism software (version 8) was used to generate graphs.<br>Image J software (version 1.8.0_172) was used for densitometry and confocal image analyses.<br>PANTHER (version 14) was used for Gene Ontology analyses.<br>Mascot (version 2.6.1) was used for identification and quantitation of proteins using Mass spectrometry data.<br>Applied Biosystems QuantStudio software (version 1.3) was used for analyses of qRT-PCR data.<br>Venn diagram was made using http://bioinformatics.psb.ugent.be/webtools/Venn/.<br>ImageStudioLite software (version 5.0) was used for quantitation of immunoblot membranes (R-loop detection).<br>BD FACSDiva 8.0.1 software (BD Biosciences) and FlowJo (version X) were used for cell cycle analysis. |

For manuscripts utilizing custom algorithms or software that are central to the research but not yet described in published literature, software must be made available to editors and reviewers. We strongly encourage code deposition in a community repository (e.g. GitHub). See the Nature Research guidelines for submitting code & software for further information.

## Data

Policy information about availability of data

All manuscripts must include a data availability statement. This statement should provide the following information, where applicable:

- Accession codes, unique identifiers, or web links for publicly available datasets
- A list of figures that have associated raw data
- A description of any restrictions on data availability

All relevant data are available from the authors upon reasonable request. Source data are provided as a Source Data file. Raw mass spectrometry data (Supplementary Table 1) files are available for download at ftp://massive.ucsd.edu/MSV000087276/.
Uniprot database https://www.uniprot.org was used for identification of proteins using Mass spectrometry data. MassIVE database https://www.massive.ucsd.edu was used for upload of Raw mass spectrometry data.

# Field-specific reporting

Please select the one below that is the best fit for your research. If you are not sure, read the appropriate sections before making your selection.

☒ Life sciences ☐ Behavioural & social sciences ☐ Ecological, evolutionary & environmental sciences

For a reference copy of the document with all sections, see nature.com/documents/nr-reporting-summary-flat.pdf

# Life sciences study design

All studies must disclose on these points even when the disclosure is negative.

| | |
|---|---|
| Sample size | Sample sizes were chosen according to accepted standards in the field. Sample size was not pre-determined using statistics tolls. As indicated in the figure legends, minimal size of analyzed biological samples was "3". For immunofluorescence experiments, at least 60-100 cells per individual sample/individual experiment were counted for statistical analyses. Statistical analysis (as described in respective figure legends) was used to calculate statistical significance of obtained results. The individual p-values are indicated in figures or in figure legends. |
| Data exclusions | No data were excluded. |
| Replication | All experiments were repeated at least 3 times, unless stated otherwise in figure legends. The main observations were reproduced in different human cell lines. Sample size and number of independent experiments are stated in figure legends. |
| Randomization | We had a limited number of biological samples. The analysis was self-normalized to the sample, so randomization of samples would not be a relevant method. In addition, the same cell culture was always split before all the various treatments were performed. |
| Blinding | As the analysis required comparisons against a known controls and knockdown targets were selected for their likely relevance to the biological pathway, blinding would not provide much reduction of potential bias in the analysis. However, performance and analyses of experiments were independently conducted by co-authors. |

# Reporting for specific materials, systems and methods

We require information from authors about some types of materials, experimental systems and methods used in many studies. Here, indicate whether each material, system or method listed is relevant to your study. If you are not sure if a list item applies to your research, read the appropriate section before selecting a response.

### Materials & experimental systems

| n/a | Involved in the study |
|---|---|
| ☐ | ☒ Antibodies |
| ☐ | ☒ Eukaryotic cell lines |
| ☒ | ☐ Palaeontology and archaeology |
| ☒ | ☐ Animals and other organisms |
| ☒ | ☐ Human research participants |
| ☒ | ☐ Clinical data |
| ☒ | ☐ Dual use research of concern |

### Methods

| n/a | Involved in the study |
|---|---|
| ☒ | ☐ ChIP-seq |
| ☐ | ☒ Flow cytometry |
| ☒ | ☐ MRI-based neuroimaging |

## Antibodies

| | |
|---|---|
| Antibodies used | Antibodies Source Dilution<br>SETX (WB) Bethyl Labs, Cat#A301-104A 1:1000<br>XRN2 (H-3) (IP) Santa Cruz, Cat#sc365258 5 µg/200µg extract |

XRN2 (WB) Bethyl Labs,  Cat#A301-103A 1:1000
GAPDH (WB) Santa Cruz,  Cat#sc47724 1:5000
TRF1-78 (IP, WB) Santa Cruz,  Cat#sc56807 1:100 (WB), 5µg/200µg extract (IP)
Cyclin A (WB) Santa Cruz,  Cat#sc271682 1:2000
Geminin (WB) Santa Cruz,  Cat#FL-209 1:2000
TRF2 (WB) Bethyl Labs,  Cat#A300-796A 1:1000
TRF2 (ChIP grade) (IP) Abcam,  Cat#ab13579 2µg/200µg extract
S9.6 (slot blot) Millipore,  Cat#MABE1095 1:500
ssDNA (slot blot) Millipore,  Cat#MAB3034 1:250
RAP1 (IP, WB) Santa Cruz,  Cat#sc53434 1:100 (WB), 3µg/200µg extract (IP)
GST (WB) Bethyl Labs,  Cat#A190-122A 1:2000
rabbit IgG (IP) Santa Cruz,  Cat#sc2027 3µg/200µg extract
mouse IgG (IP) Millipore,  Cat#12-371 3µg/200µg extract
Cyclin E (WB) BD Pharmingen,  Cat#BDB551159 1:2000
TIN2 (IP) Abcam,  Cat#ab197894 2µg/200µg extract
TIN2 (WB) Origene,  Cat#CF809776 1:1000
TPP1 (IP) Abcam,  Cat#ab112050 2µg/200µg extract
TPP (WB) Bethyl,  Cat#A303-069A 1:1000
53BP1 (WB) Novus Biologicals,  Cat#NB100-304 1:5000
POT1 (WB) Biovision,  Cat#A1916 1:500
POT1 (IP) Proteintech,  Cat#10581-1-AP 3µg/200µg extract
anti-HA.11 (WB) Biolegend,  Cat#16B12 1:2000
p27 (WB) BD Biosciences,  Cat#BD610241 1:1000
pH3(Ser10) (WB) Millipore,  Cat#06-570 1:5000
BRCA1 SD118 (IP, WB) Millipore,  Cat#OP107 1:500 (WB), 3µg/200µg extract (IP)
BRCA2 (WB) Bethyl Labs,  Cat#A300-005A 1:1000
Vinculin (G-11) (WB) Santa Cruz,  Cat#sc-55465 1:5000
BARD1 (WB) Bethyl Labs,  Cat#A300-263A 1:1000
MDC1 (WB) Bethyl Labs,  Cat#A300-052A 1:1000
RPA34 (WB) Millipore,  Cat#NA19L 1:1000
GFP (WB) Novus Biologicals,  Cat#NB600308 1:2000
RNAse H1 (WB) Genetex,  Cat#GTX117624 1:500
V5-tag (D3H8Q) (WB) Cell Signaling,  Cat#13202 1:5000
HDAC1 (WB) Cell Signaling,  Cat#34589 1:5000
BRCA1 (IF) Millipore,  Cat#07-434 1:800
pChk1S345 (IF) Life Technologies,  Cat#MA515145 1:500
pATR1989 (IF) Genetex,  Cat#GTX128145 1:1000
TRF2 (IF) Abcam,  Cat#ab13579 1:1000
PML (IF) Abcam,  Cat#ab96051 1:500
HA (IF) Biolegend,  Cat#16B12 1:1000
γH2AX (IF) Millipore,  Cat#05-636 1:300
S9.6 (DRIP) DFCI Monoclonal antibody Core 2.5µg/15µg sheared DNA
RNA Polymerase II (ChIP) Biolegend,  Cat#8WG16 4µg/20µg sheared DNA
RNA-PolII-(S2) (ChIP) Bethyl, Cat#A300-654A 1µg/20µg sheared DNA
RNA-PolII-(S5) (ChIP) Bethyl, Cat#A304-408A 1µg/20µg sheared DNA
BRCA1 (ChIP) Bethyl, Cat#A300-000A 1µg/20µg sheared DNA
DNMT3b (ChIP) Novus Biologicals, Cat#NB300516 2µg/20µg sheared DNA
DNMT1 (ChIP) Novus Biologicals, Cat#NB100-56519 1µg/20µg sheared DNA
H3K27me3 (ChIP) Abcam, Cat#ab6002 2µg/20µg sheared DNA
H3K9me3 (ChIP) Abcam, Cat#8898 2µg/20µg sheared DNA
H3K4me3 (ChIP) Abcam, Cat#ab8580 2µg/20µg sheared DNA
H4K16ac (ChIP) Millipore, Cat#07329MI 5µg/20µg sheared DNA
H4 (ChIP) Millipore, Cat#04858MI 5µg/20µg sheared DNA
H3 (ChIP) Abcam, Cat#ab1791 2µg/20µg sheared DNA

Validation

The specificity of mouse anti-BRCA1 antibody (validated by the manufacturer for IP and WB) was confirmed by WB of CRISPR-modified and hairpin inducible BRCA1-depleted HME cells (Fig.1i, Supplementary Fig. 2c-d) as well as by BRCA1 IP (Fig.1a, Fig.1e). rabbit anti-BARD1 antibody was confirmed by the manufacturer as suitable for WB and was used for confirmation of BRCA1 status in CRISPR-modified BRCA1-depleted HME cells in Supplementary Fig. 2c.

Antibodies directed against mouse-anti XRN2 (Santa Cruz), mouse anti-TRF2 (Abcam), rabbit anti-TPP1 (Abcam), mouse anti-RAP1 (Santa Cruz), mouse anti-TRF1-78 (Santa Cruz), and rabbit IgG as well as mouse IgG are all validated by the manufacturer as suitable for IP analyses. Specificity of signals was confirmed by comparison against IPs using nonspecific antibodies (mouse/rabbit IgG). In addition, rabbit anti-XRN2, rabbit anti-TRF2, and rabbit anti-TPP antibodies, validated by the manufacturer for WB, were used to confirm specificity of IP analyses since they target a different epitope. Respective data can be found in Fig. 1a, 1d-e, Supplementary Fig.1a, 1g.
Rabbit anti-SETX antibody was used as a positive control for BRCA1 pulldown (Fig.1e) and is validated by the manufacturer as suitable for WB. Also, this antibody has been previously used in Livingston lab, and respective data can be found in "Hatchi E, Skourti-Stathaki K, et al. BRCA1 recruitment to transcriptional pause sites is required for R-loop-driven DNA damage repair. Mol Cell. 2015 Feb 19;57(4):636-647. doi: 10.1016/j.molcel.2015.01.011. PMID: 25699710; PMCID: PMC4351672."

Antibodies directed against DNA repair proteins, rabbit anti-BRCA2, rabbit anti-MDC1, mouse anti-RPA34, rabbit anti-53BP1, are validated by the manufacturer for WB analyses. Rabbit anti-BRCA2 was used as a positive control for GST experiments (Supplementary Fig. 1i). MDC1, RPA34, and 53BP1 antibodies were used for RNA/R-loop affinity assays, and respective signals were compared to negative controls (Fig.2b-d).

Rabbit anti-HDAC1, mouse anti-vinculin, and mouse anti-GAPDH antibodies were used for WB loading and fractionation (HDAC1 nuclear soluble and vinculin cytoplasmic) control purposes, all of which are validated by the manufacturer for WB: Fig.1i, 2g, 4e; Supplementary Fig. 1b, 2c-d, 3e, 3k, 3p, 4d, 4i, 4k, 4o, 5d, 5h-i, 6a, 6i.

Antibodies used for slot blot analysis of R-loops are mouse anti-S9.6 and mouse-anti ssDNA, validated by the manufacturer. Specificity of S9.6 signals was confirmed by treatment with RNAse A - Supplementary Fig. 1j.

Antibodies directed at BRCA1 protein tags (HA and GST) are validated by the manufacturer for WB analysis. Specificity of signals were confirmed by comparison of transfected vs non-transfected cells and negative controls such as beads only:
mouse anti-HA.11 - Fig.4e, Supplementary Fig. 5d, h-i, Supplementary Fig. 6i
rabbit anti-GST - Fig.4b-d, Supplementary Fig. 1h-i, 5a-b

Rabbit antibody against RNAse H1 is validated by the manufacturer for WB analysis, and was used in "Arora, R., Lee, Y., Wischnewski, H. et al. RNaseH1 regulates TERRA-telomeric DNA hybrids and telomere maintenance in ALT tumour cells. Nat Commun 5, 5220 (2014). https://doi.org/10.1038/ncomms6220". Antibodies directed at RNAse H1 protein tags (GFP and V5) are validated by the manufacturer for WB analysis. Specificity of signals were confirmed by the presence of signal in transfected vs non-transfected samples:
rabbit anti-RNAse H1 - Fig.1i, Supplementary Fig.3e, Supplementary Fig.4d
rabbit anti-GFP - Supplementary Fig.1l-m
rabbit anti-V5-tag - Fig.1i, Supplementary Fig.3e, Supplementary Fig.4d, Supplementary Fig.6a

Antibodies used for cell cycle analyses were recommended by people in the field and are validated by the manufacturer for WB experiments. Cell synchronization experiments were performed confirming cell cycle-dependent expression of relevant proteins by WB:
mouse anti-Cyclin A (S phase)- Supplementary Fig.1b
rabbit anti-Geminin (S phase)- Figure 2g, Supplementary Fig.1b, Supplementary Fig.3k, 3p
rabbit anti-pH3(Ser10) (M phase) - Figure 2g, Supplementary Fig.1b, Supplementary Fig.3k, 3p
mouse anti-p27 (senescence marker) - Supplementary Fig.3k
mouse anti-Cyclin E (G1 and G1/S)- Figure 2g, Supplementary Fig.1b, Supplementary Fig.3k, 3p

Antibodies used for immunofluorescence analyses are validated by the manufacturer for IF experiments, and respective IF images can be found:
rabbit anti-BRCA1 - Supplementary Fig.1c, Supplementary Fig.3f-i
rabbit anti-pChk1S345 - Fig. 5b, Supplementary Fig.6b, g
rabbit anti-pATR1989 - Fig. 5a, 5d, Supplementary Fig.6c
mouse anti-TRF2 - Fig. 5a-b, d, Supplementary Fig.6g
mouse anti-PML - Supplementary Fig.3i, Supplementary Fig.6b-c
mouse anti-HA - Supplementary Fig.5c
mouse anti-γH2AX - Fig.1j, Supplementary Fig.2e-g

Specificity of S9.6 antibody, purified in DFCI Monoclonal antibody Core, was confirmed by increased qRT-PCR signal over beads (values can be found in Source data file - calculations).
ChIP antibodies used in this study were validated for ChIP analysis by the manufacturer, and their specificity was confirmed by increased qRT-PCR signal over beads (values can be found in Source data file - calculations). Respective data can be found:
mouse anti-RNA Polymerase II - Fig.3e-f
rabbit anti-RNA-PolII-(S2) - Fig.3e-f
rabbit anti-RNA-PolII-(S5) - Fig.3e-f
rabbit anti-BRCA1 - Fig.1b, g; Fig.2k
rabbit anti-DNMT3b - Fig.3k, Supplementary Fig.4n
mouse anti-DNMT1 - Supplementary Fig.4m-n
mouse anti-H3K27me3 - Fig.3g-i
rabbit anti-H3K9me3 - Fig.3g-i
rabbit anti-H3K4me3 - Fig.3g-i
rabbit anti-H4K16ac - Fig.3g-i
rabbit anti-H4 - Fig.3g-i
rabbit anti-H3 - Fig.3g-i

Validation of rabbit anti-TIN2 (Abcam, not validated for IP by the manufacturer) and rabbit anti-POT1 (Proteintech, validated for IP by the manufacturer) antibodies for IP analyses are shown in the Peer Review File (siRNA-depleted cells and nonspecific IgG antibody as negative controls). In addition, mouse anti-TIN2 (Origene, validated by the manufacturer for WB) and rabbit anti-POT1 (Biovision, validated by the manufacturer for WB) antibodies were used to confirm specificity of IP analyses since they target different epitopes. Respective IP and WB analyses can be found in Fig.1a, d-e and Supplementary Fig.1a, g.
POT1 antibody was also previously validated for IP in "Singh U, Maturi V, Jones RE, Paulsson Y, Baird DM, Westermark B. CGGBP1 phosphorylation constitutes a telomere-protection signal. Cell Cycle. 2014;13(1):96-105. doi: 10.4161/cc.26813. Epub 2013 Oct 23. PMID: 24196442; PMCID: PMC3925742".

# Eukaryotic cell lines

Policy information about cell lines

| | |
|---|---|
| Cell line source(s) | HeLa, U2OS, T98G, U2OS DR-GFP cells were obtained by the laboratory from ATCC.<br>Human mammary epithelial cells (HMECs) were generated by the Weinberg laboratory (MIT) (BRCA1/FANCD2/BRG1-Driven DNA Repair Stabilizes the Differentiation State of Human Mammary Epithelial Cells. Mol Cell. 2016;63(2):277-292. doi:10.1016/j.molcel.2016.05.038). |

| | |
|---|---|
| | Non-immortalized and telomerase immortalized HME cells are gift from Dr. Shailja Pathania (UMass Boston) (Pathania, S., Bade, S., Le Guillou, M. et al. BRCA1 haploinsufficiency for replication stress suppression in primary cells. Nat Commun 5, 5496 (2014). https://doi.org/10.1038/ncomms6496).<br>S9.6 hybridoma cell line was purchased from ATCC (HB-8730; purified in DFCI Core). |
| Authentication | HeLa, U2OS, T98G, U2OS DR-GFP, S9.6 hybridoma cell lines were authenticated by the company from which we purchased the cells.<br>Non-immortalized and telomerase immortalized HME cells (gift from Dr. Shailja Pathania) were fully characterized in the following publication (Pathania, S., Bade, S., Le Guillou, M. et al. BRCA1 haploinsufficiency for replication stress suppression in primary cells. Nat Commun 5, 5496 (2014). https://doi.org/10.1038/ncomms6496).<br>shBRCA1 HMECs were fully characterized by other members of the lab and published in several studies, including a few of the Livingston lab.<br>HMECs showed expression of mammary epithelial markers (BRCA1/FANCD2/BRG1-Driven DNA Repair Stabilizes the Differentiation State of Human Mammary Epithelial Cells. Mol Cell. 2016;63(2):277-292. doi:10.1016/j.molcel.2016.05.038). |
| Mycoplasma contamination | All cell lines were routinely tested and are negative for mycoplasma. |
| Commonly misidentified lines<br>(See ICLAC register) | No commonly misidentified cell lines were used. |

# Flow Cytometry

## Plots

Confirm that:

☒ The axis labels state the marker and fluorochrome used (e.g. CD4-FITC).

☒ The axis scales are clearly visible. Include numbers along axes only for bottom left plot of group (a 'group' is an analysis of identical markers).

☒ All plots are contour plots with outliers or pseudocolor plots.

☒ A numerical value for number of cells or percentage (with statistics) is provided.

## Methodology

| | |
|---|---|
| Sample preparation | Cell cycle distribution was analyzed based on DNA staining using Propidium Iodide (PI) (eBioscience) according to the manufacturer's protocol. Cells were washed with cold PBS, and fixed with cold 70% ethanol overnight. The PI staining was carried out by resuspending the cells in 500µl PI/Triton X-100 solution (0.1% Triton X-100 in PBS, 0.2 mg/ml RNAse A, and PI 1:200) for 30 min at room temperature.<br>For the HR assay, cells were trypsinized and washed two times with 1xPBS prior to analysis. |
| Instrument | BD LSR Fortessa |
| Software | BD FACSDiva 8.0.1 software (BD Biosciences) and FlowJo X for cell cycle analysis. |
| Cell population abundance | 30, 000 and 10,000 cells were examined for cell cycle analysis and HR analysis, respectively. |
| Gating strategy | For DR-GFP and cell cycle experiments, doublets were removed, and further selection was based on forward and side scatter. Specificity of GFP/dsRed signals were based on comparison of negative and positive samples (untrasfected vs transfected).<br>For cell cycle experiments, cell cycle phases G1, S, and G2/M were identified based on an intensity of PI staining. |

☒ Tick this box to confirm that a figure exemplifying the gating strategy is provided in the Supplementary Information.
