# Supplementary material for: BRCA1 binds TERRA RNA and suppresses R-Loop-based telomeric DNA damage
Source: Nat Commun. 2021 Jun 10;12:3542. doi: 10.1038/s41467-021-23716-6 (PMC8192922; doi:10.1038/s41467-021-23716-6)

Figure 1j

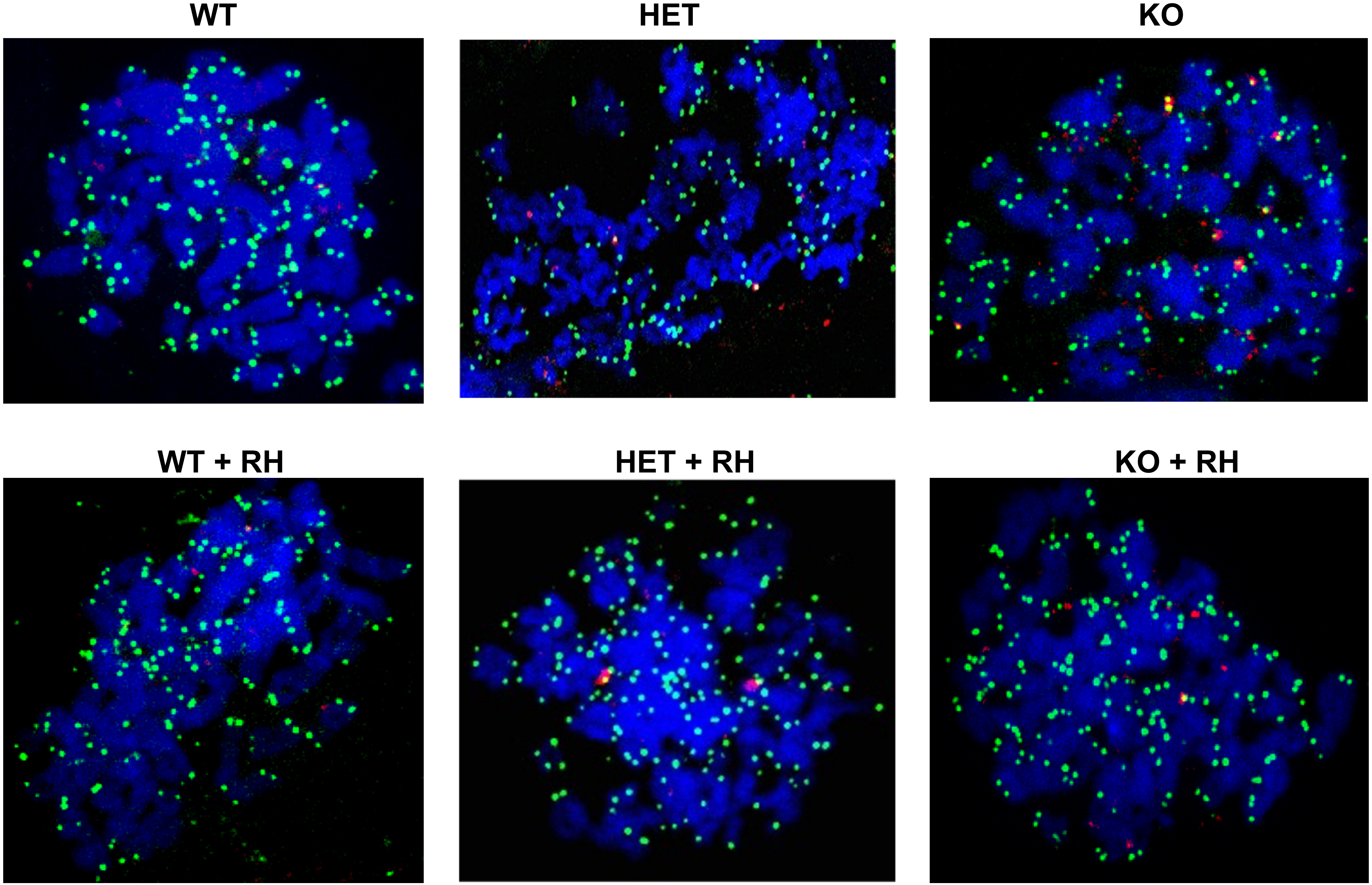

Figure 4h

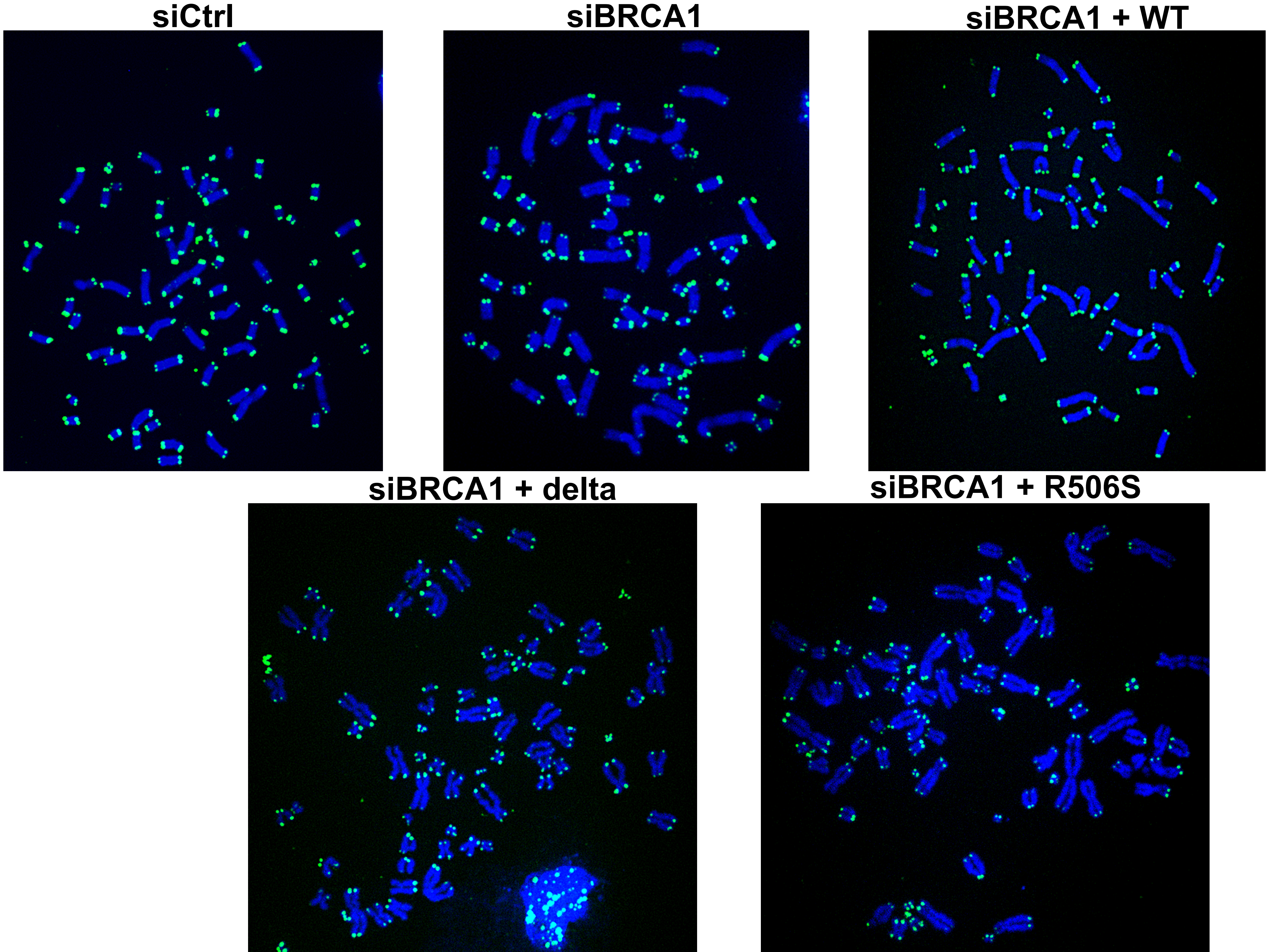

Figure 5a

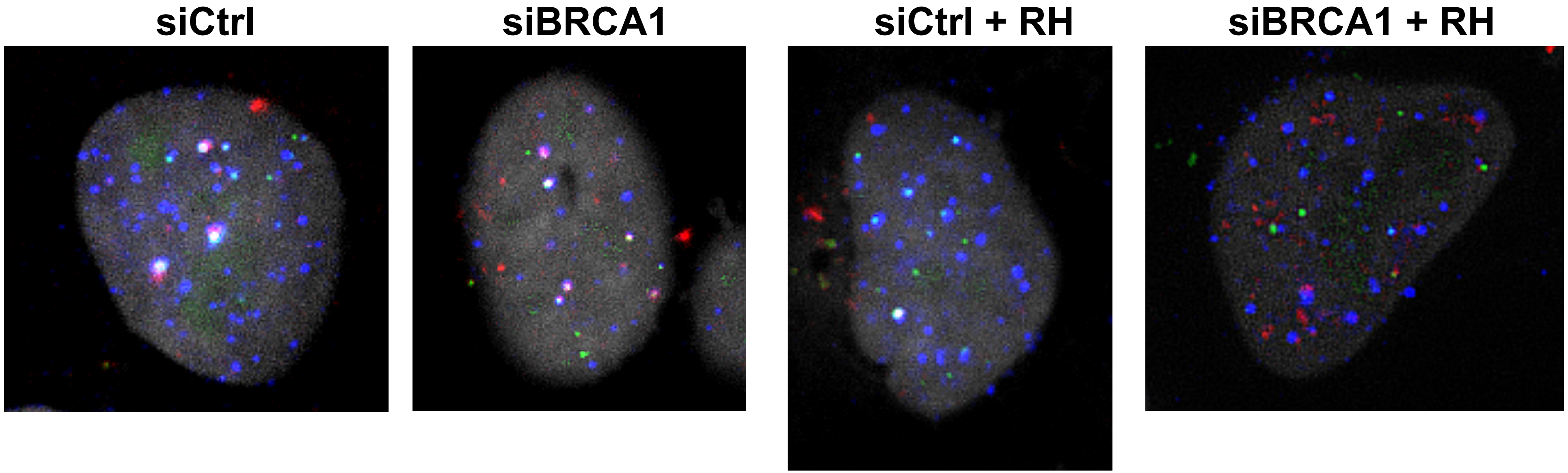

Figure 5b

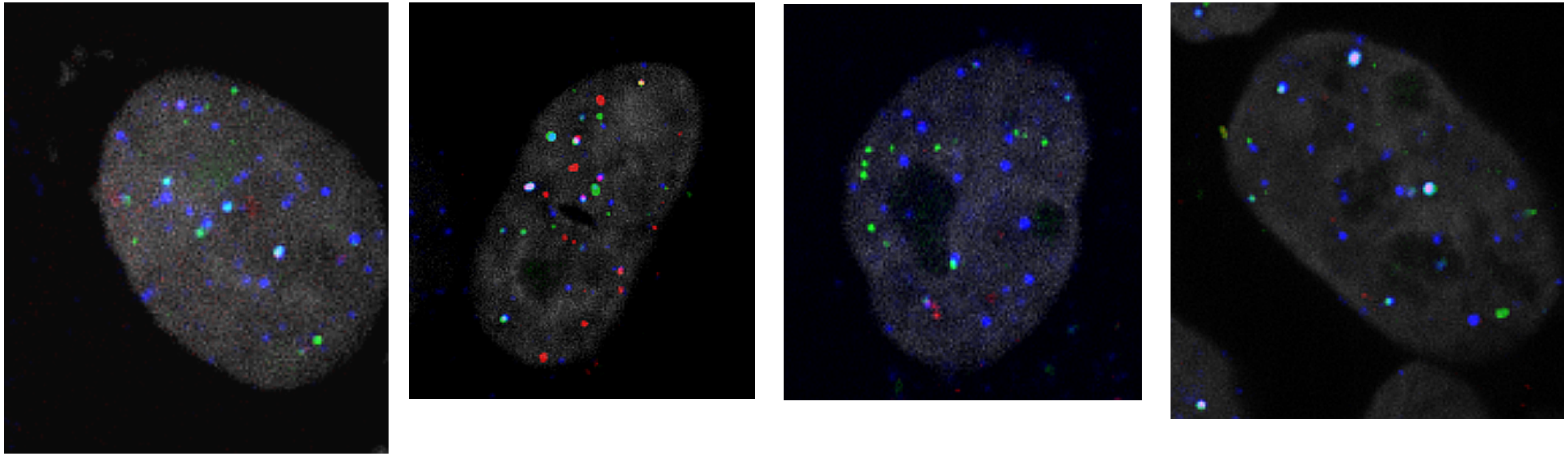

Figure 5d

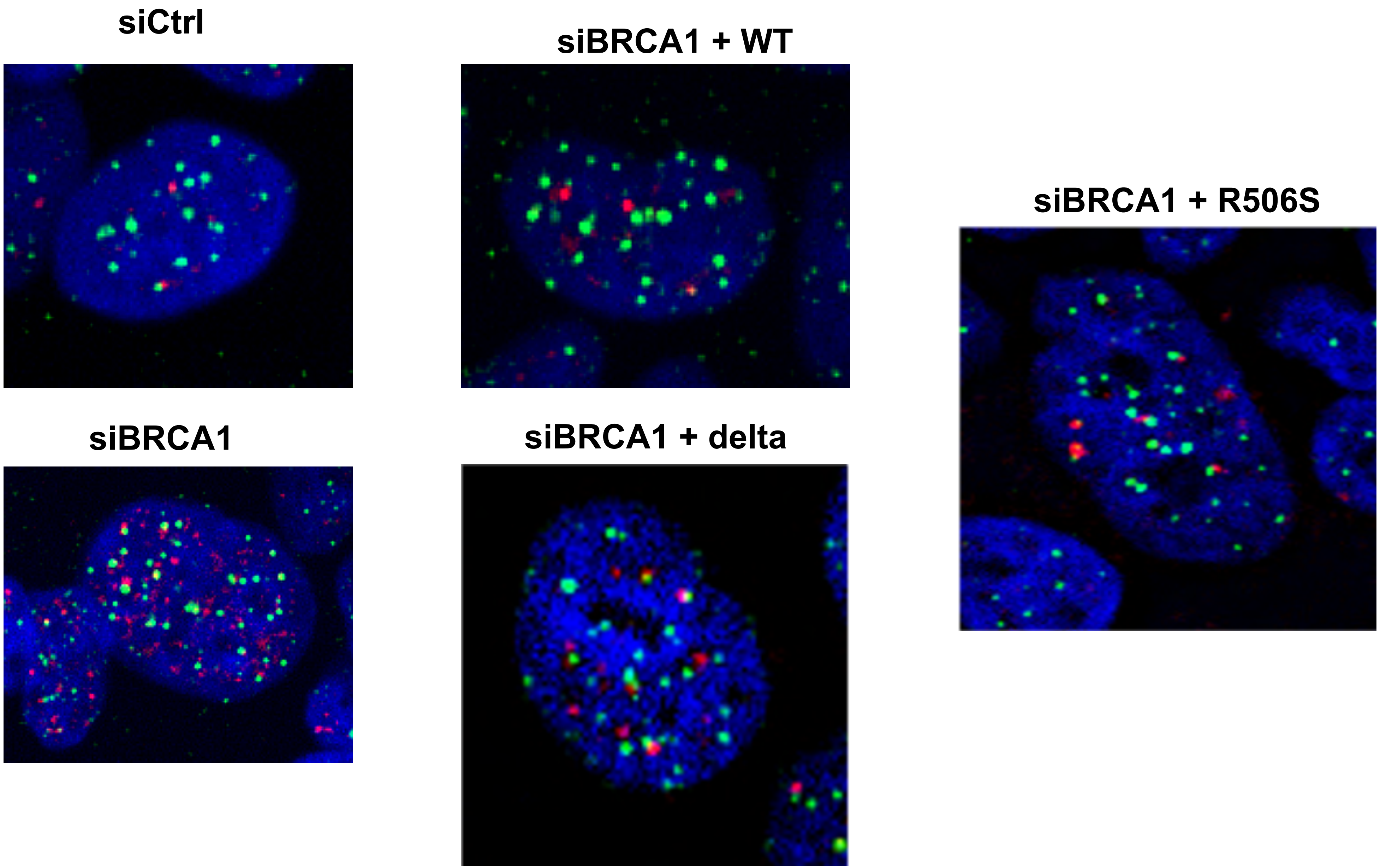

Figure 5f

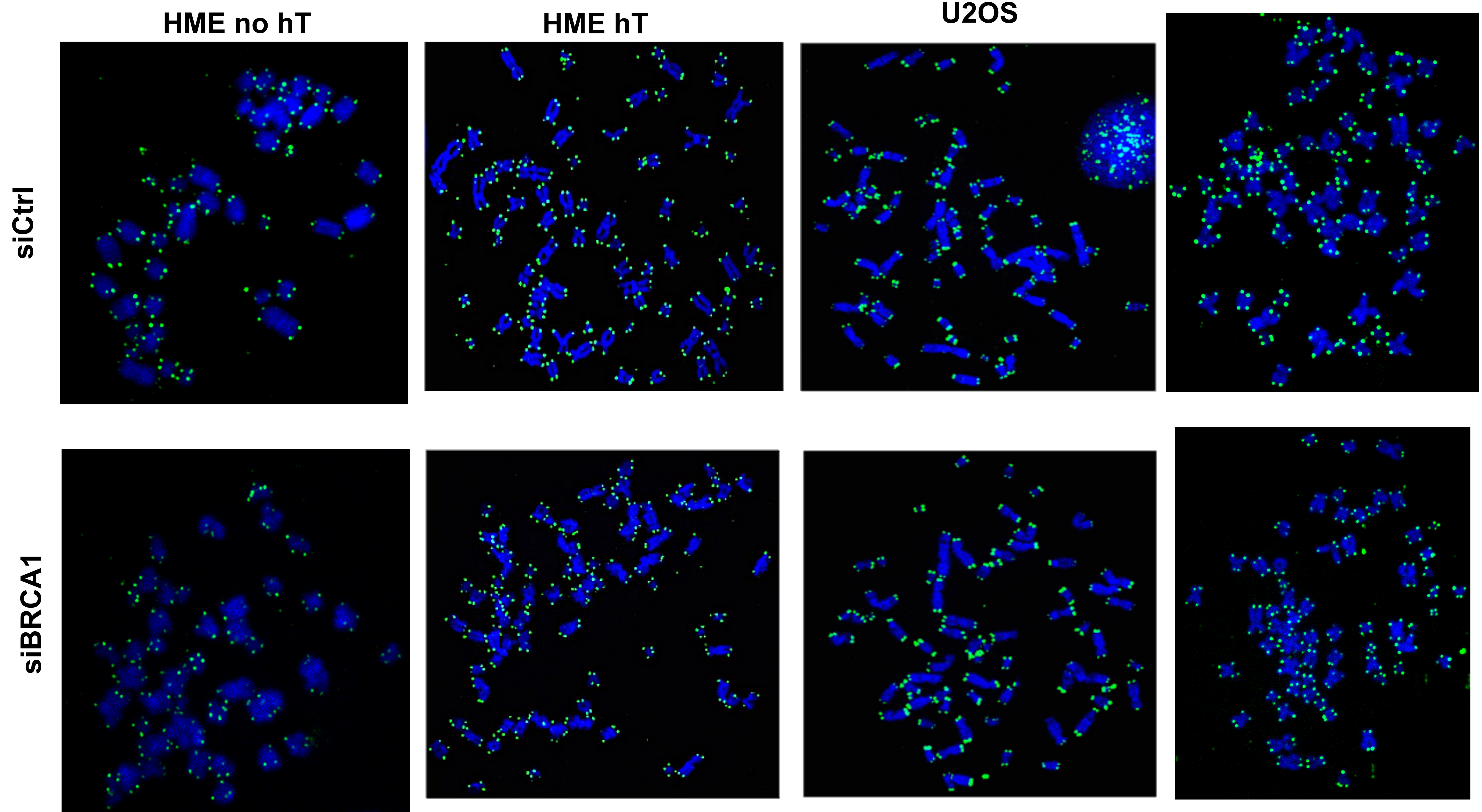

Supplementary Figure 1c

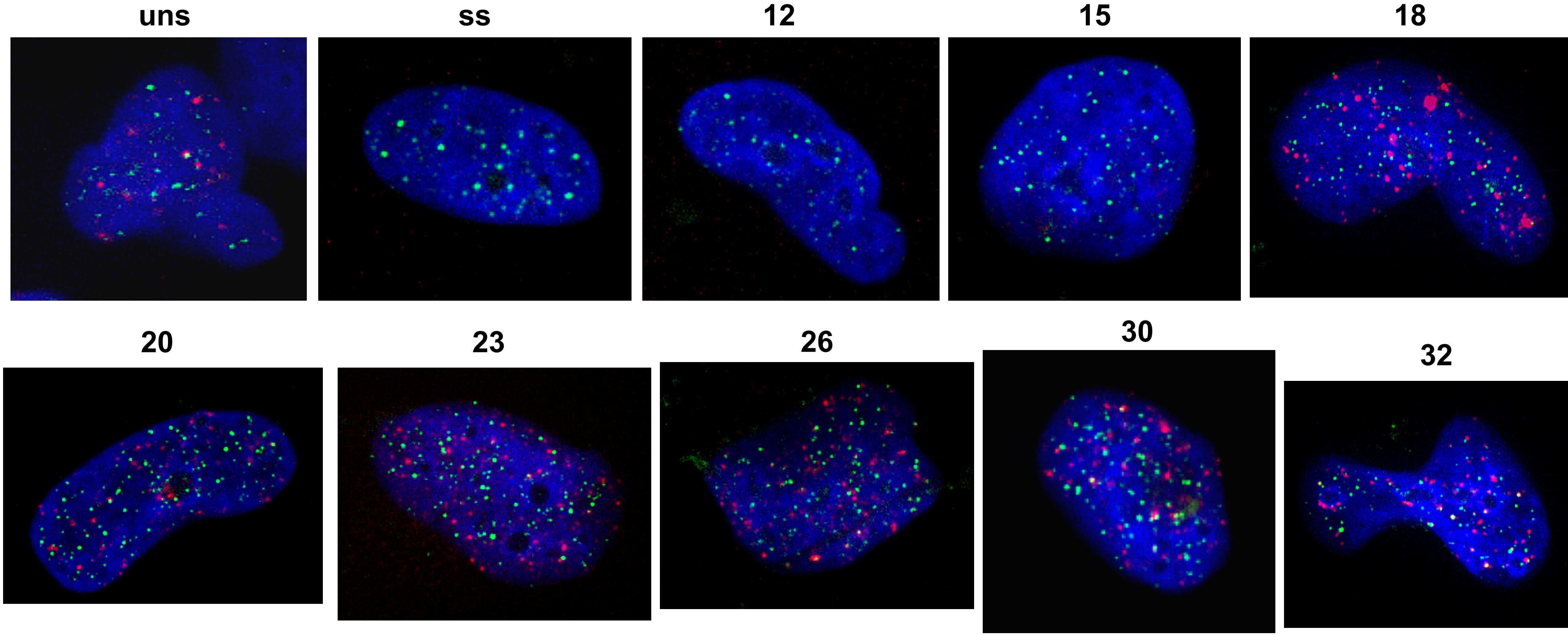

Supplementary Figure 2e

shBRCA1 | shBRCA1 + dox

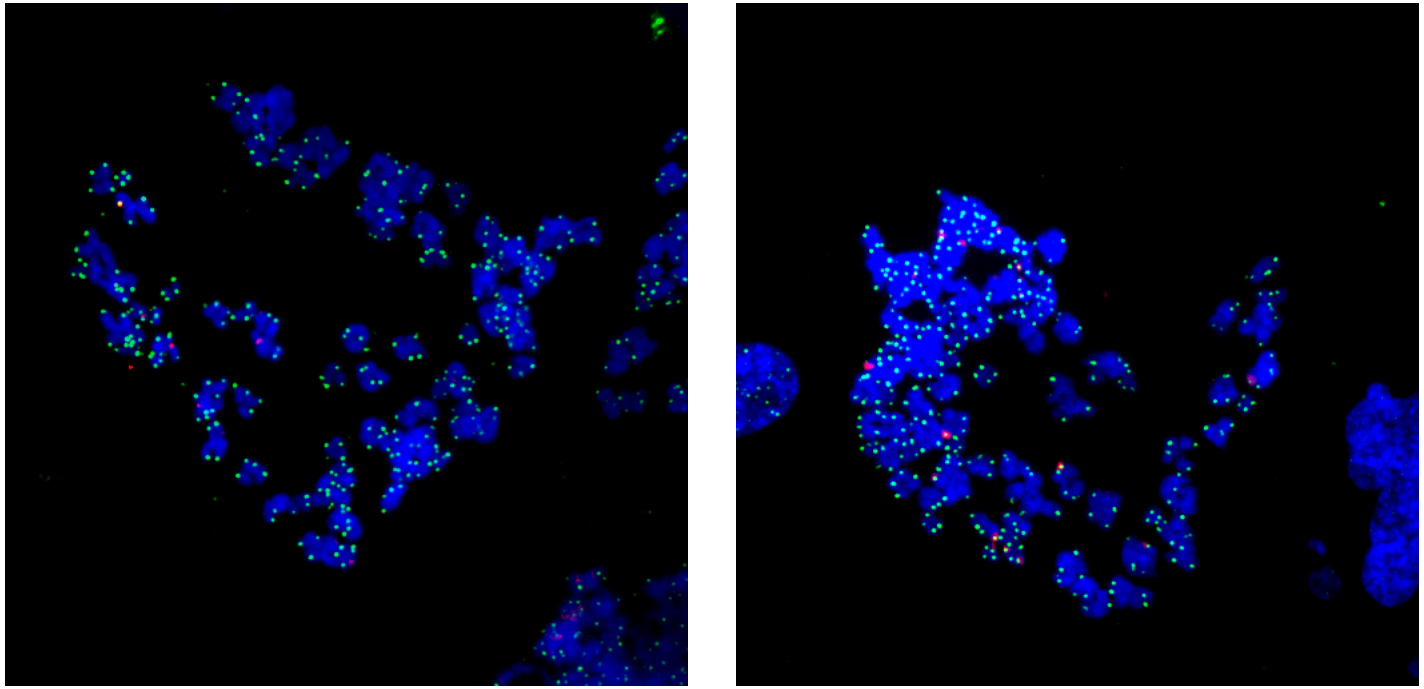

Supplementary Figure 2g

siCtrl

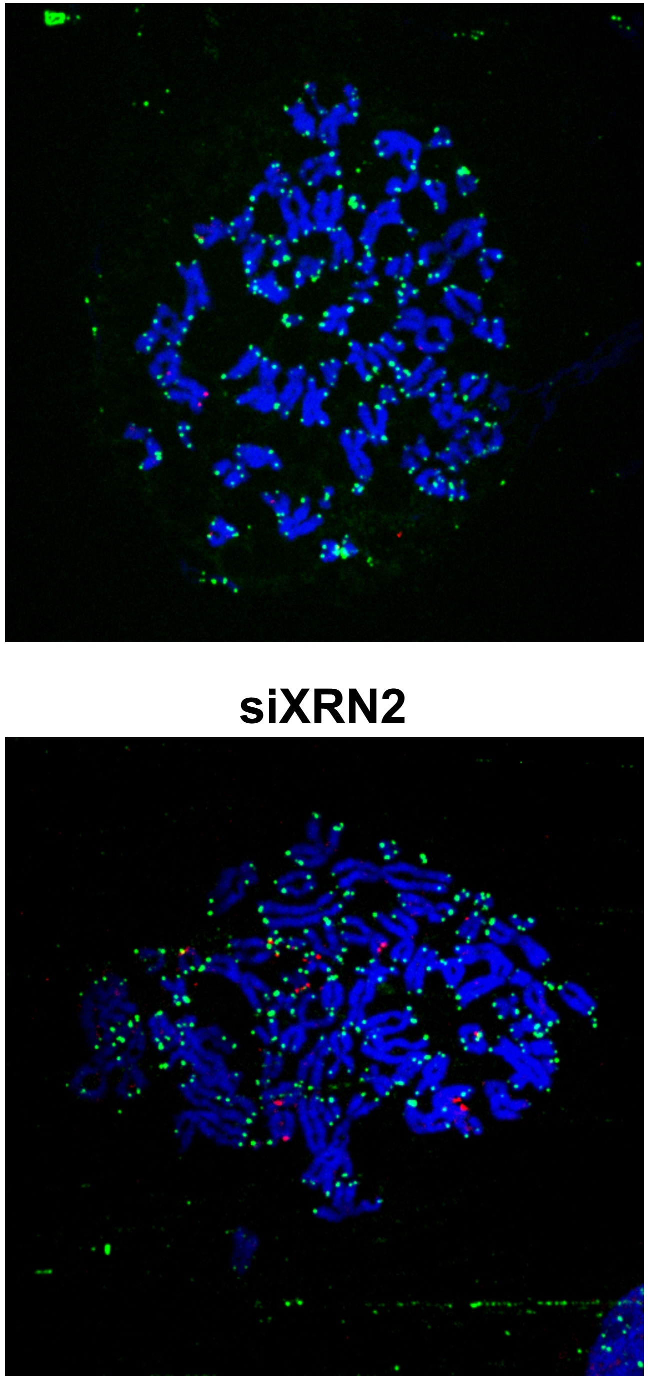

Supplementary Figure 3f

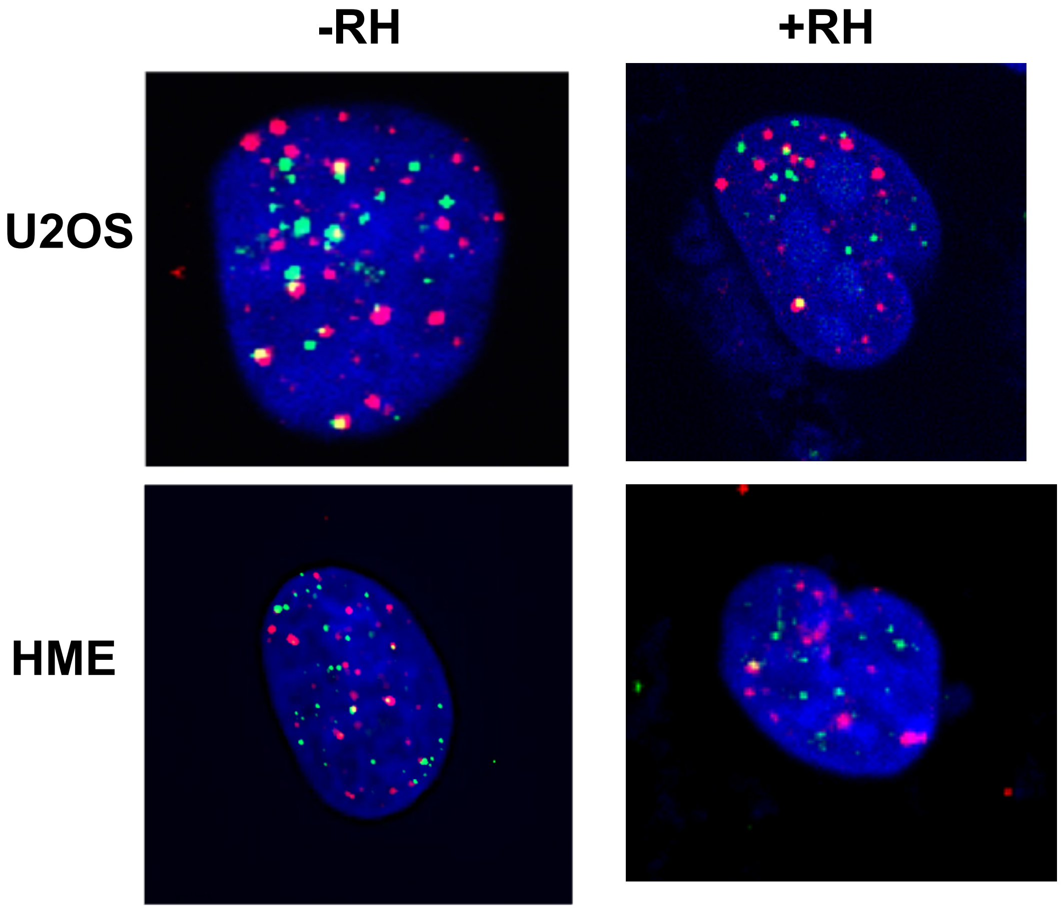

Supplementary Figure 3g

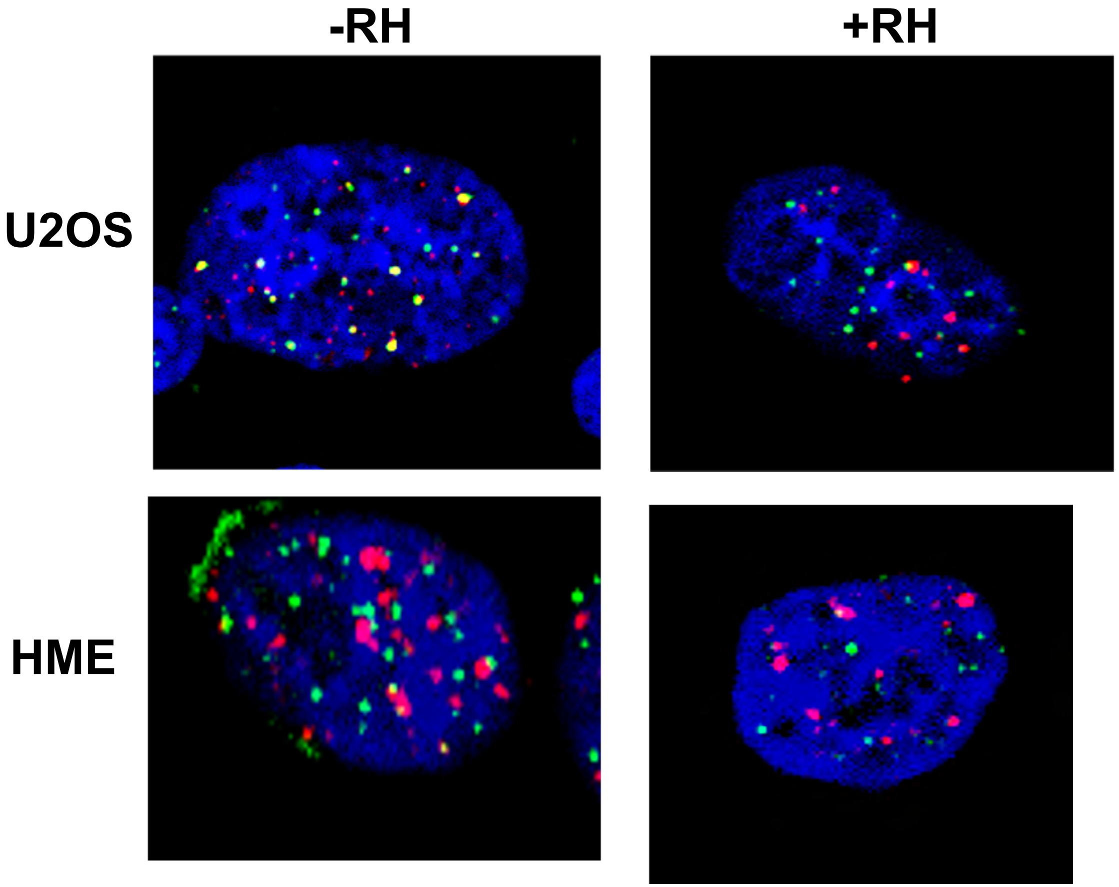

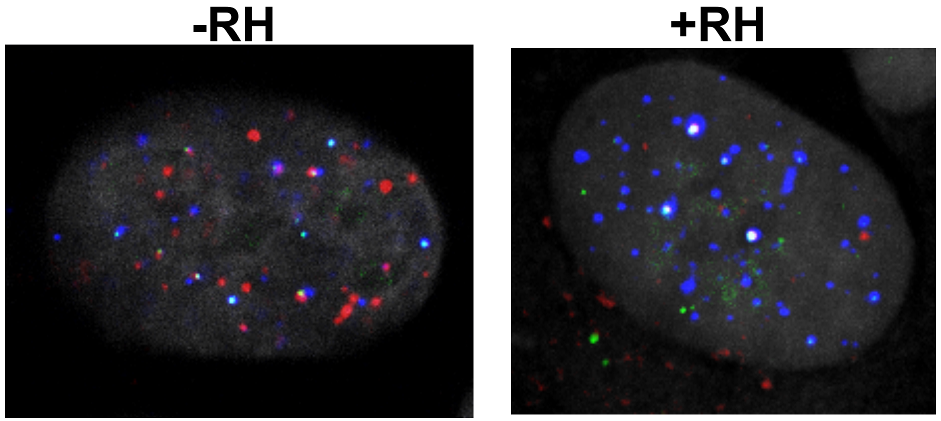

Supplementary Figure 3i

Supplementary Figure 5c

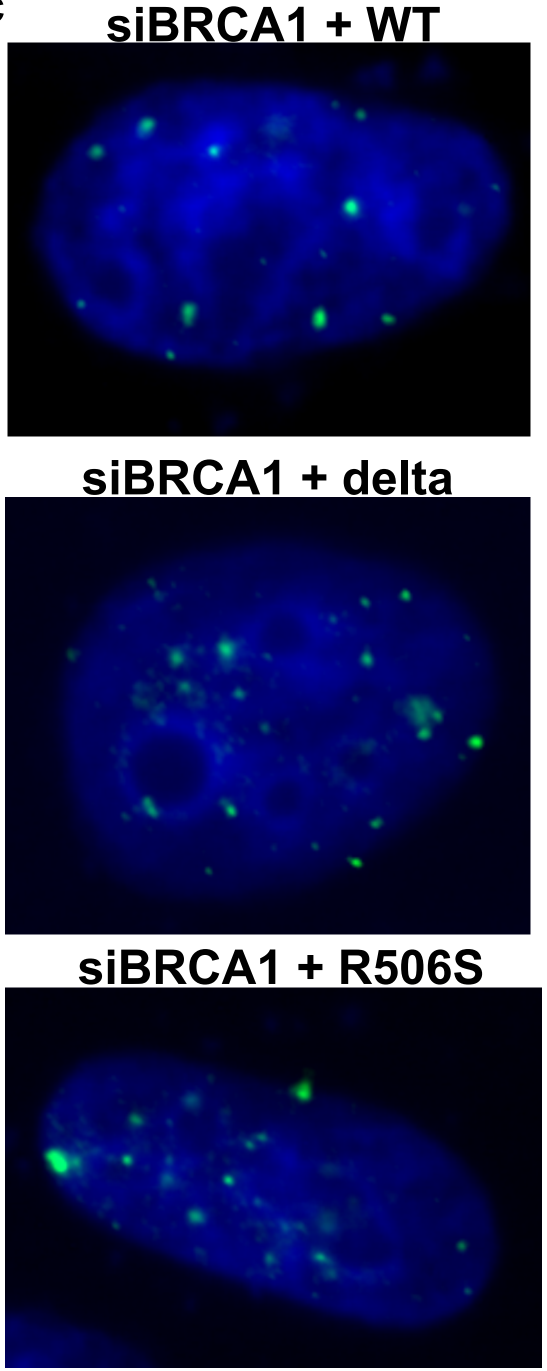

Supplementary Figure 4b

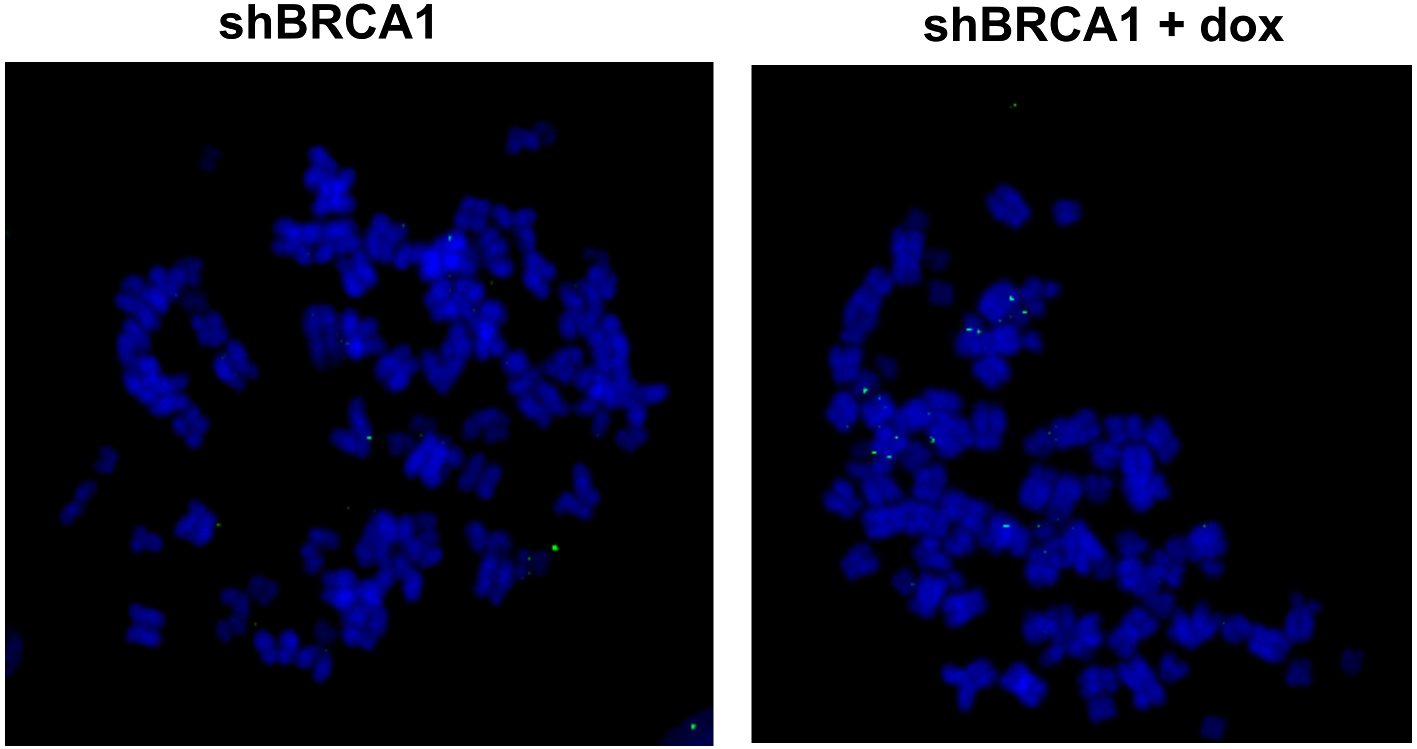

	siCtrl	siBRCA1	siCtrl + RH	siBRCA1 + RH

Supplementary Figure 6b

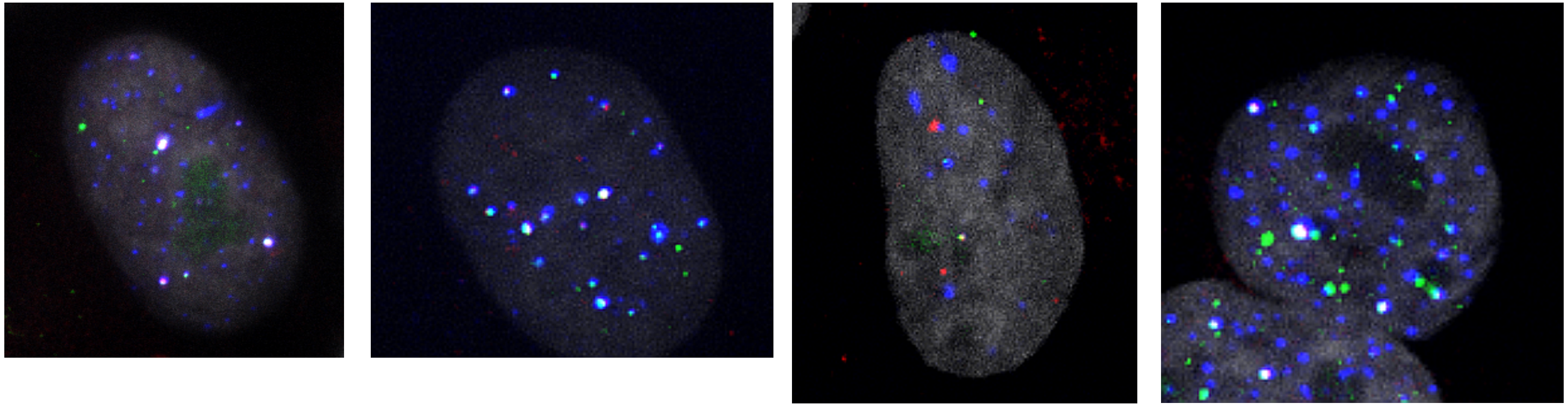

Supplementary Figure 6c

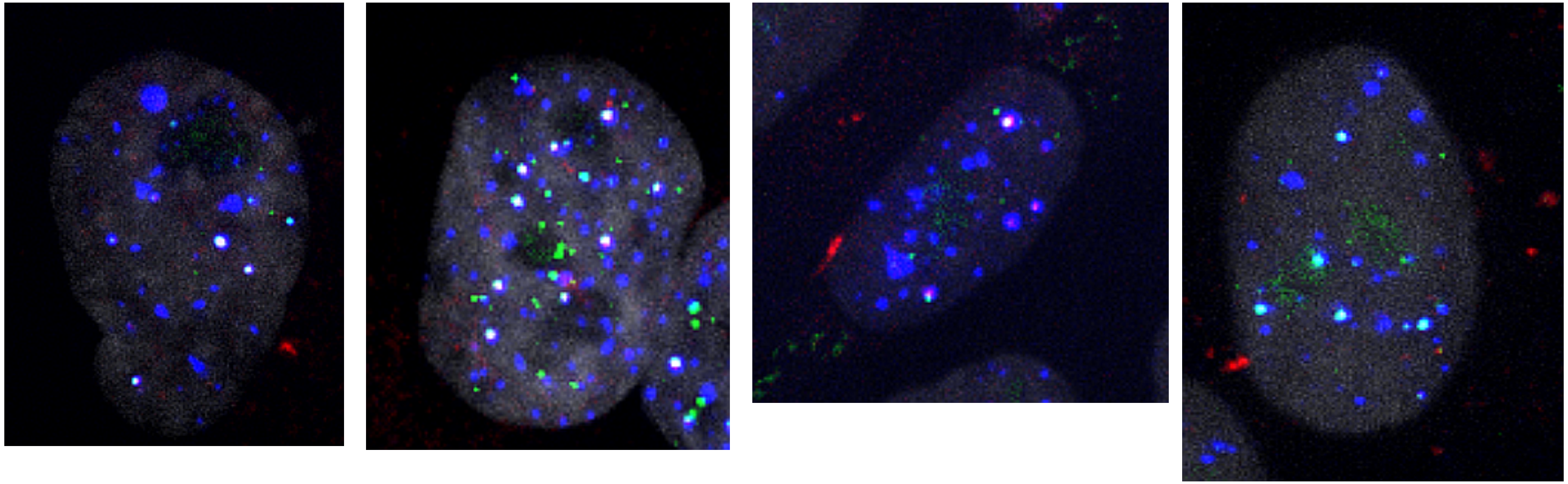

Supplementary Figure 6g

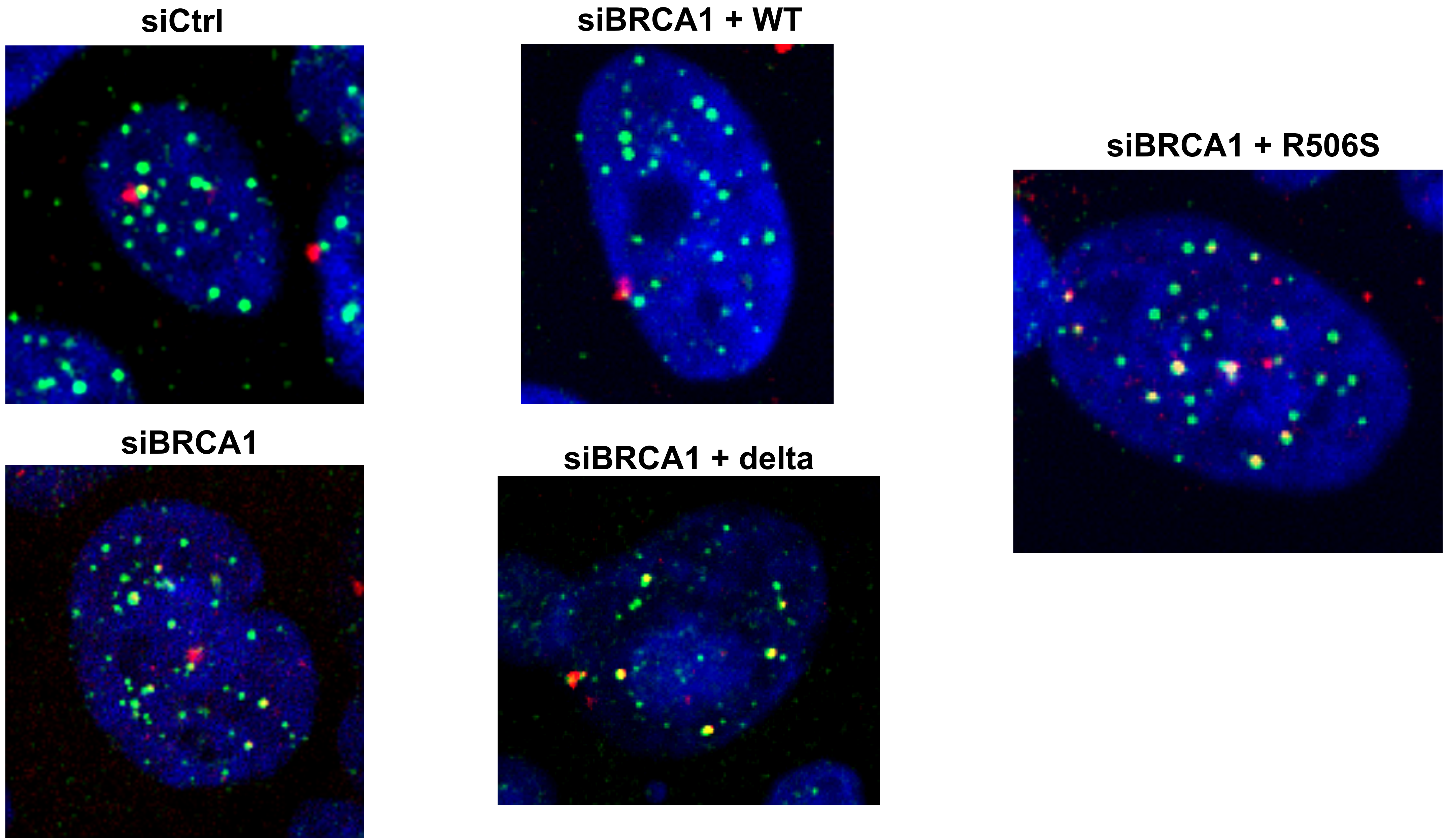

Supplement: Supplementary file 4 — Source Data [file 41467_2021_23716_MOESM4_ESM.zip › 266613_2_supp_5525004_qs7404.pdf]
