# Supplementary figures and images for: BRCA1 binds TERRA RNA and suppresses R-Loop-based telomeric DNA damage
Source: Nat Commun. 2021 Jun 10;12:3542. doi: 10.1038/s41467-021-23716-6 (PMC8192922; doi:10.1038/s41467-021-23716-6)

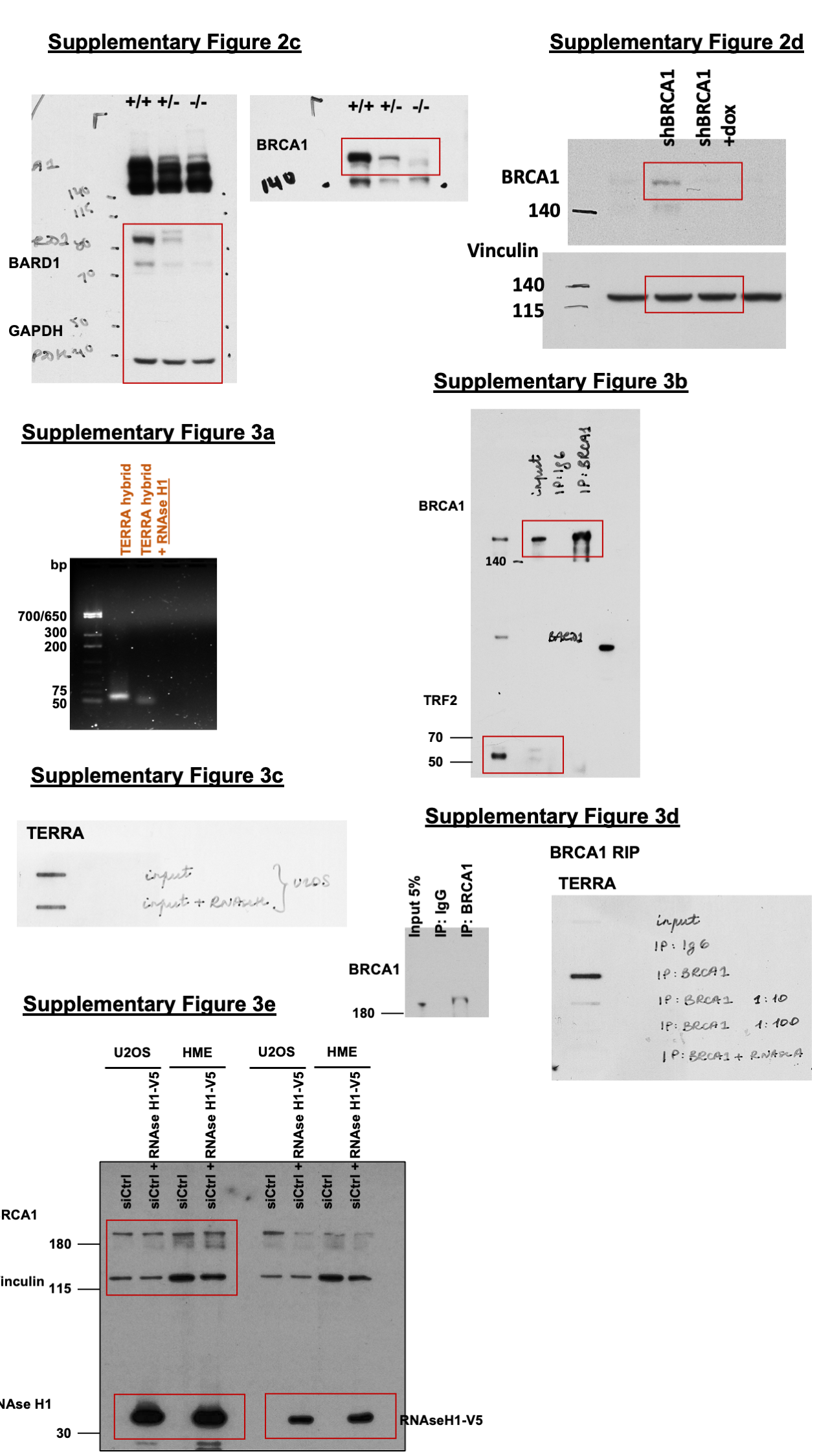

Supplement: Supplementary file 4 — Source Data [file 41467_2021_23716_MOESM4_ESM.zip › 266613_2_supp_5525009_qs7405.png]

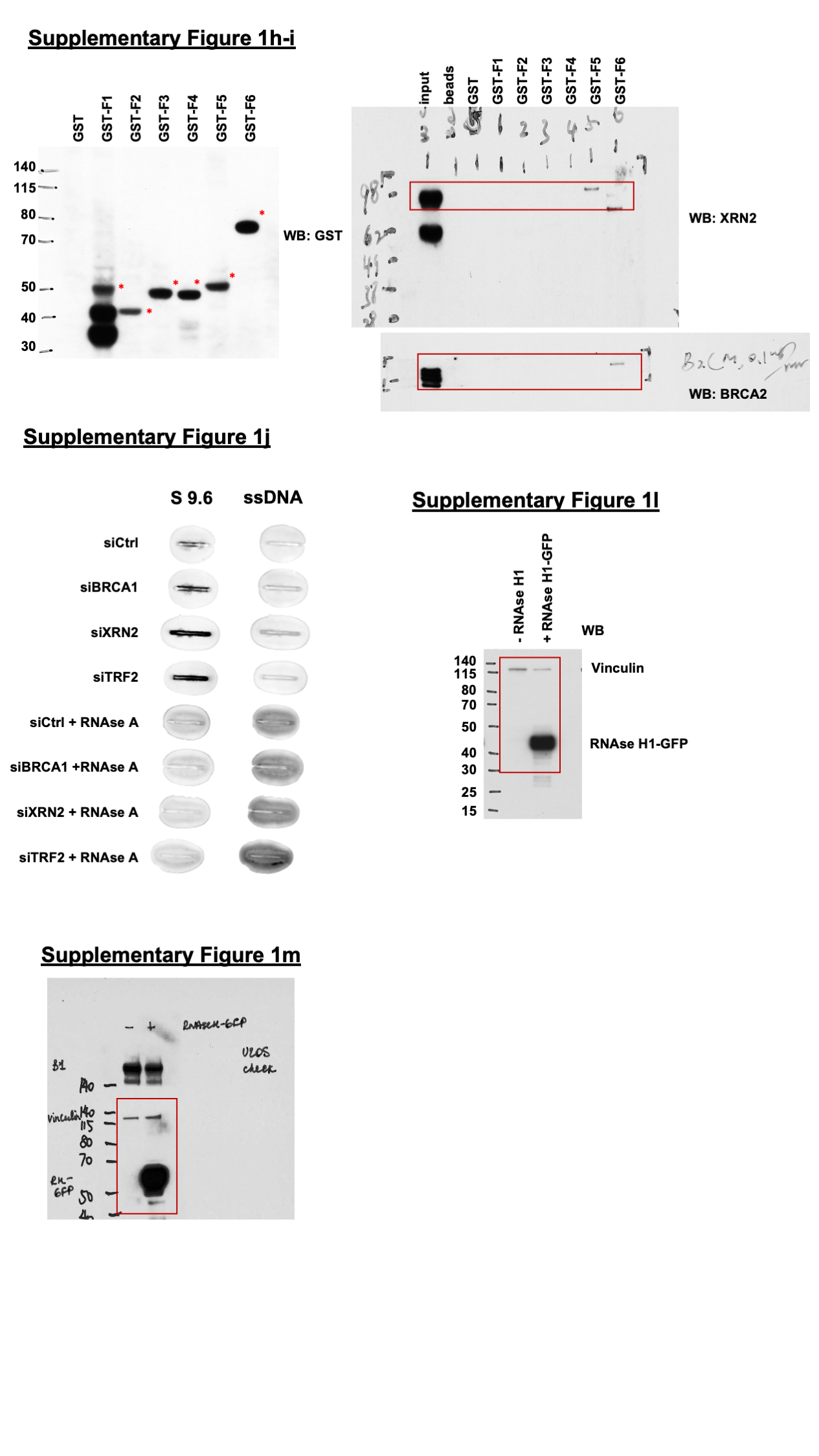

Supplement: Supplementary file 4 — Source Data [file 41467_2021_23716_MOESM4_ESM.zip › 266613_2_supp_5525010_qs7405.png]

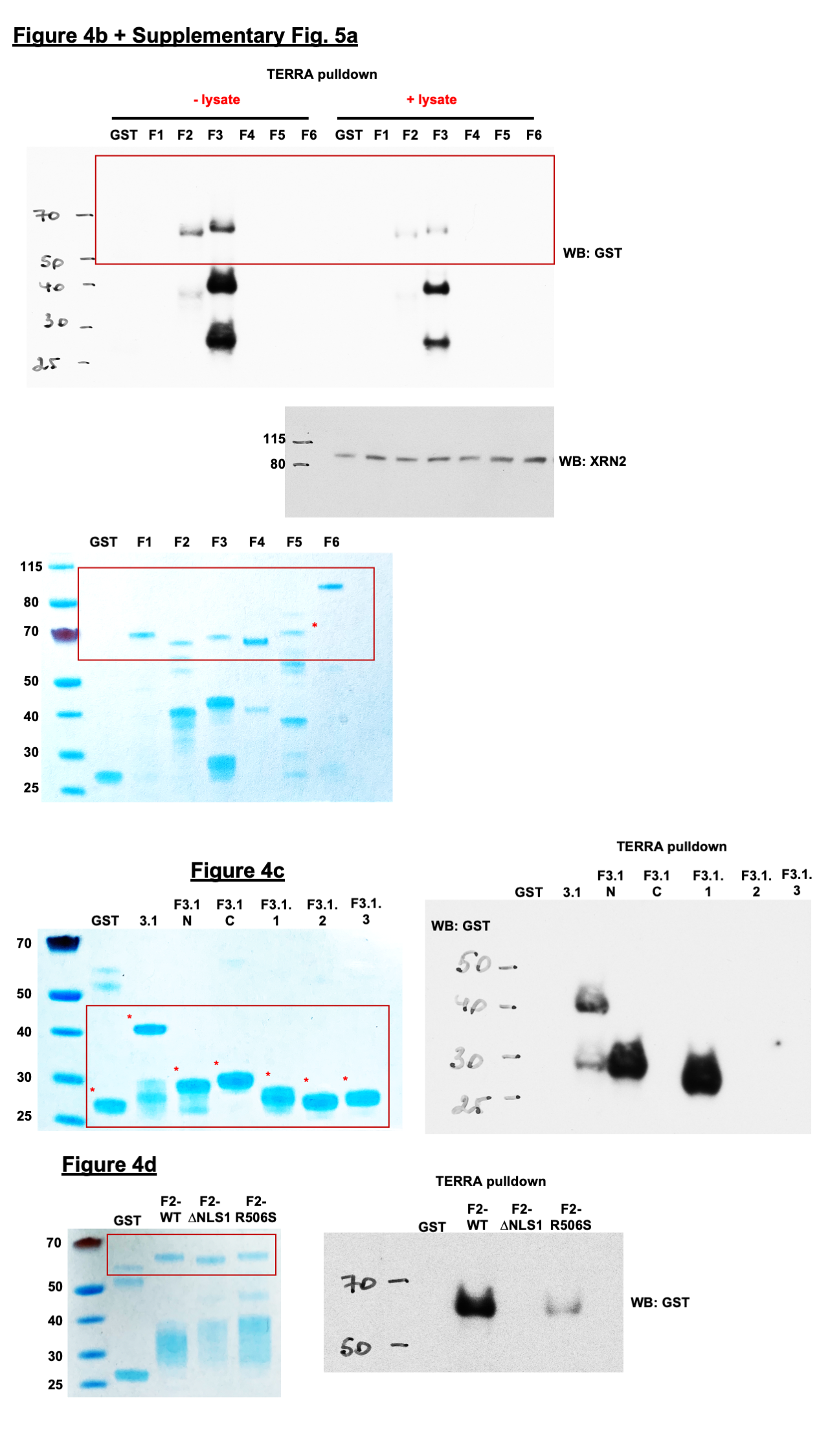

Supplement: Supplementary file 4 — Source Data [file 41467_2021_23716_MOESM4_ESM.zip › 266613_2_supp_5525011_qs7405.png]

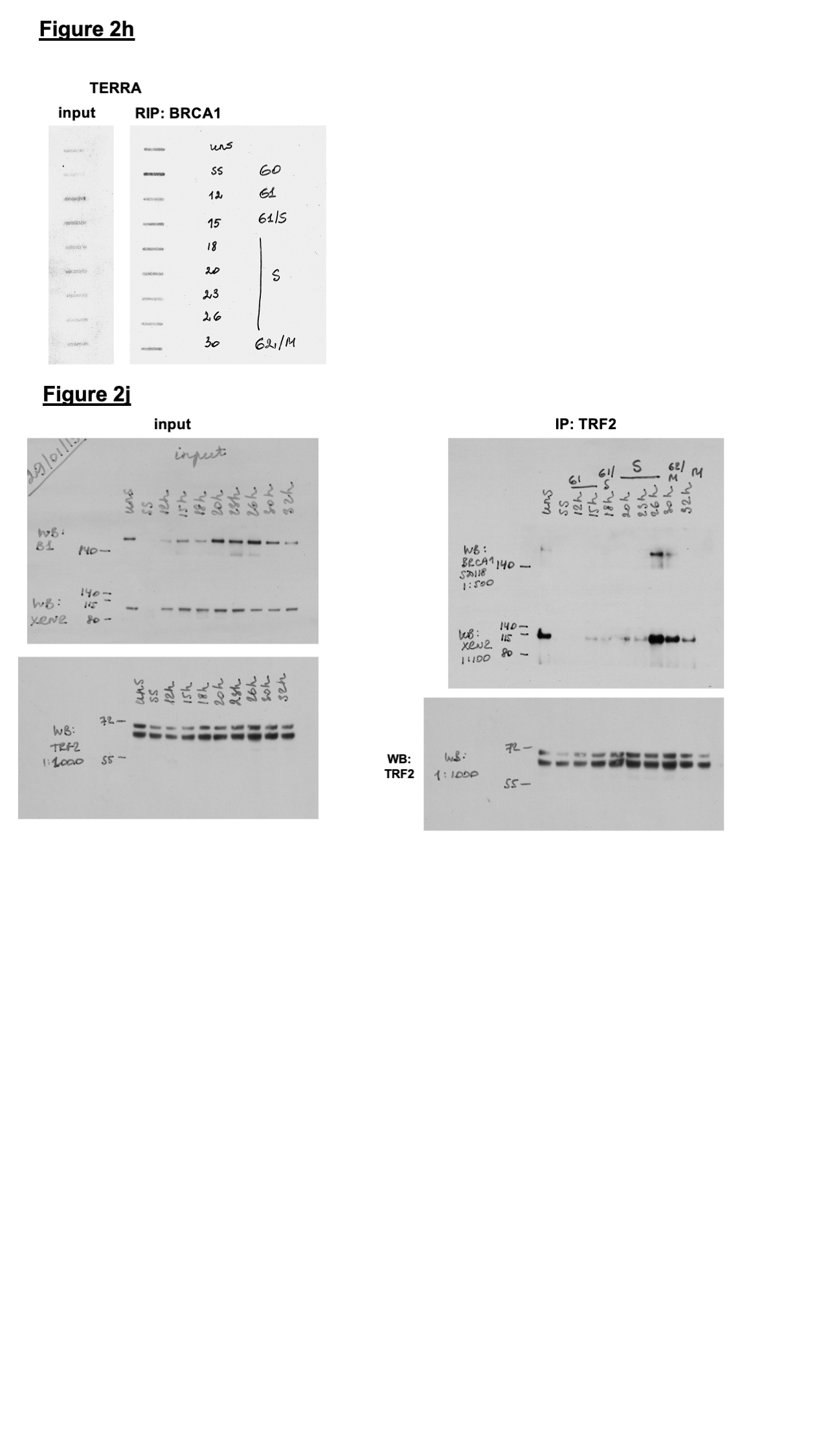

Supplement: Supplementary file 4 — Source Data [file 41467_2021_23716_MOESM4_ESM.zip › 266613_2_supp_5525012_qs7405.png]

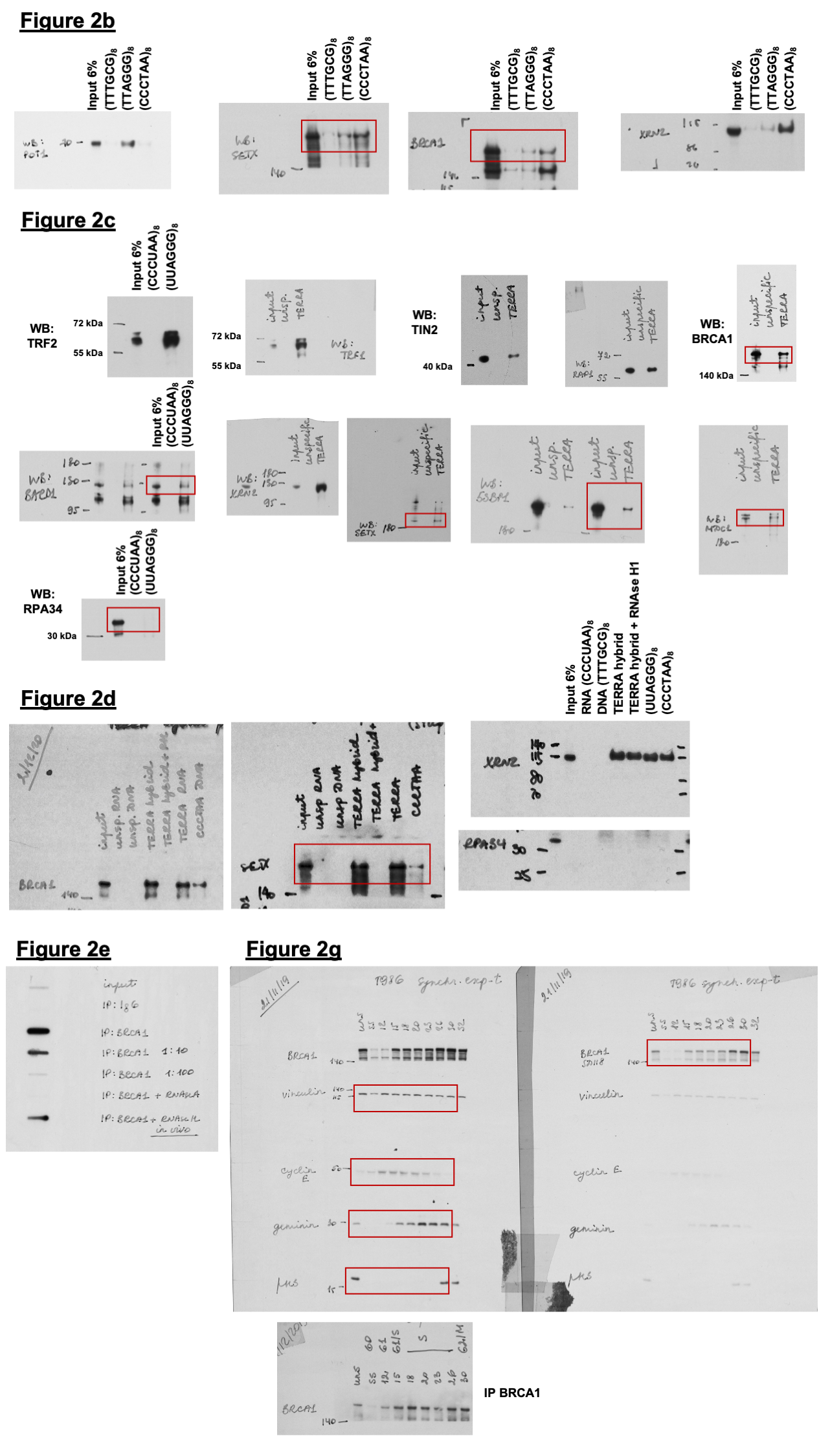

Supplement: Supplementary file 4 — Source Data [file 41467_2021_23716_MOESM4_ESM.zip › 266613_2_supp_5525013_qs7405.png]

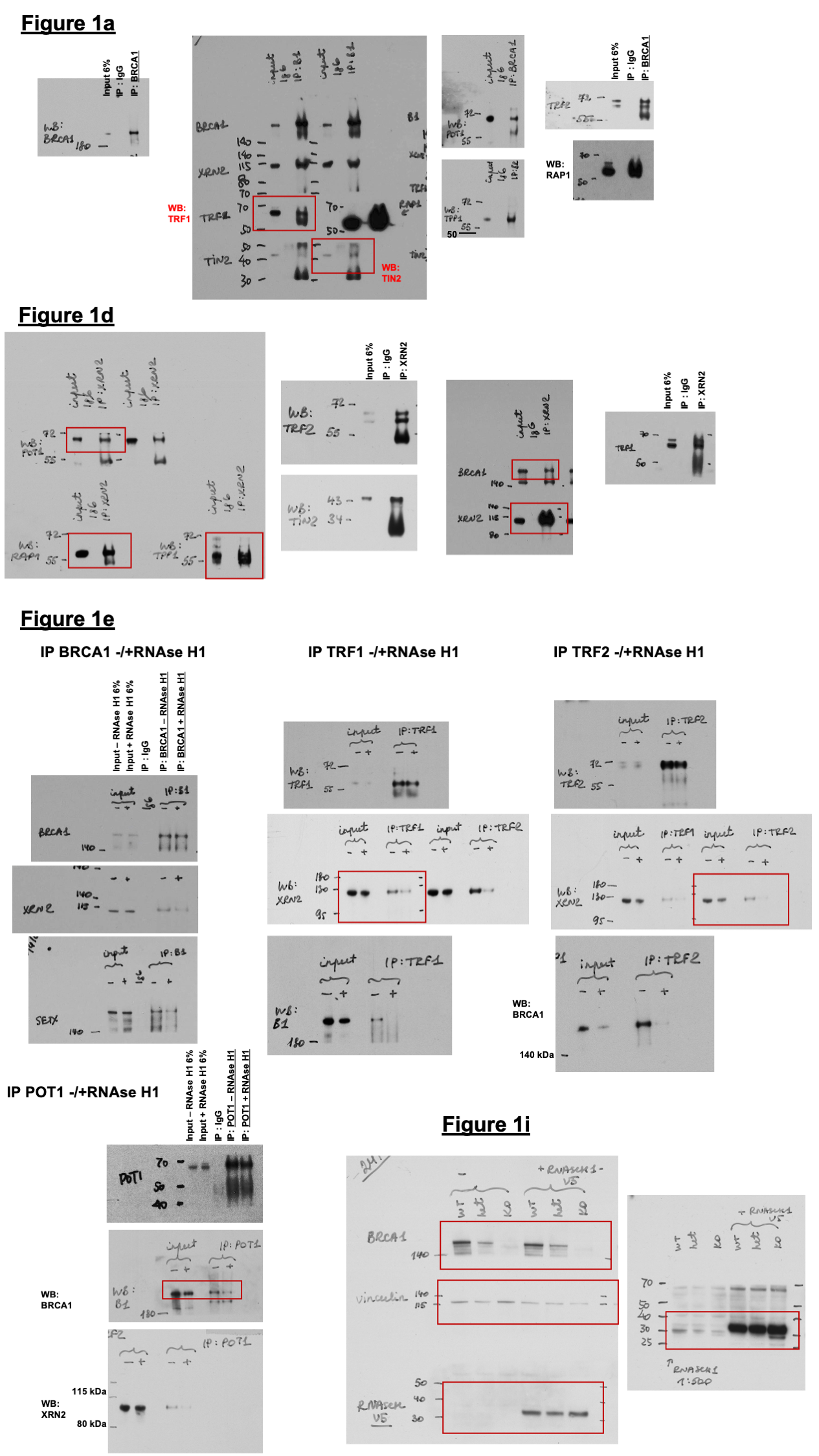

Supplement: Supplementary file 4 — Source Data [file 41467_2021_23716_MOESM4_ESM.zip › 266613_2_supp_5525014_qs7405.png]

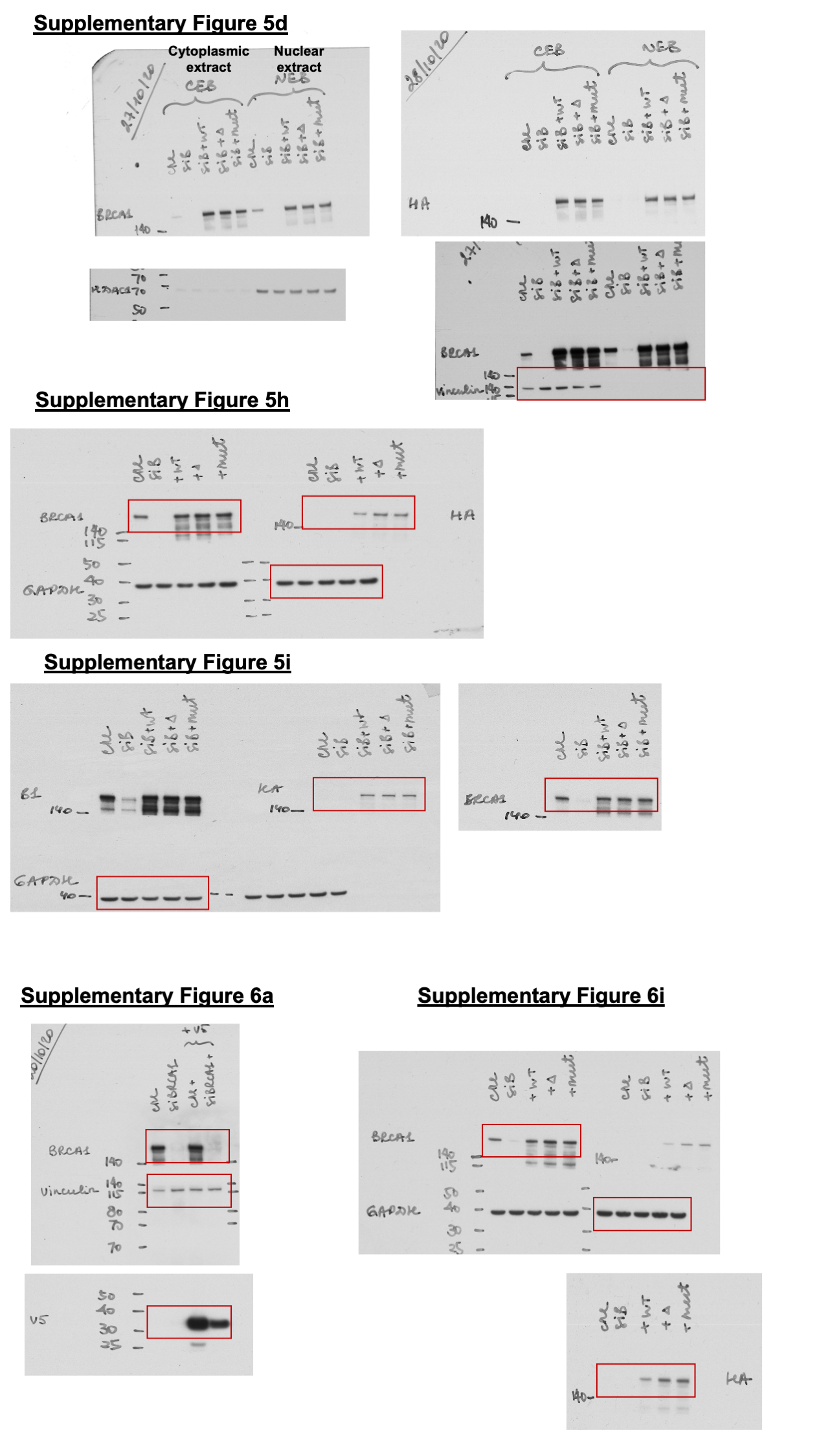

Supplement: Supplementary file 4 — Source Data [file 41467_2021_23716_MOESM4_ESM.zip › 266613_2_supp_5525015_qs7405.png]

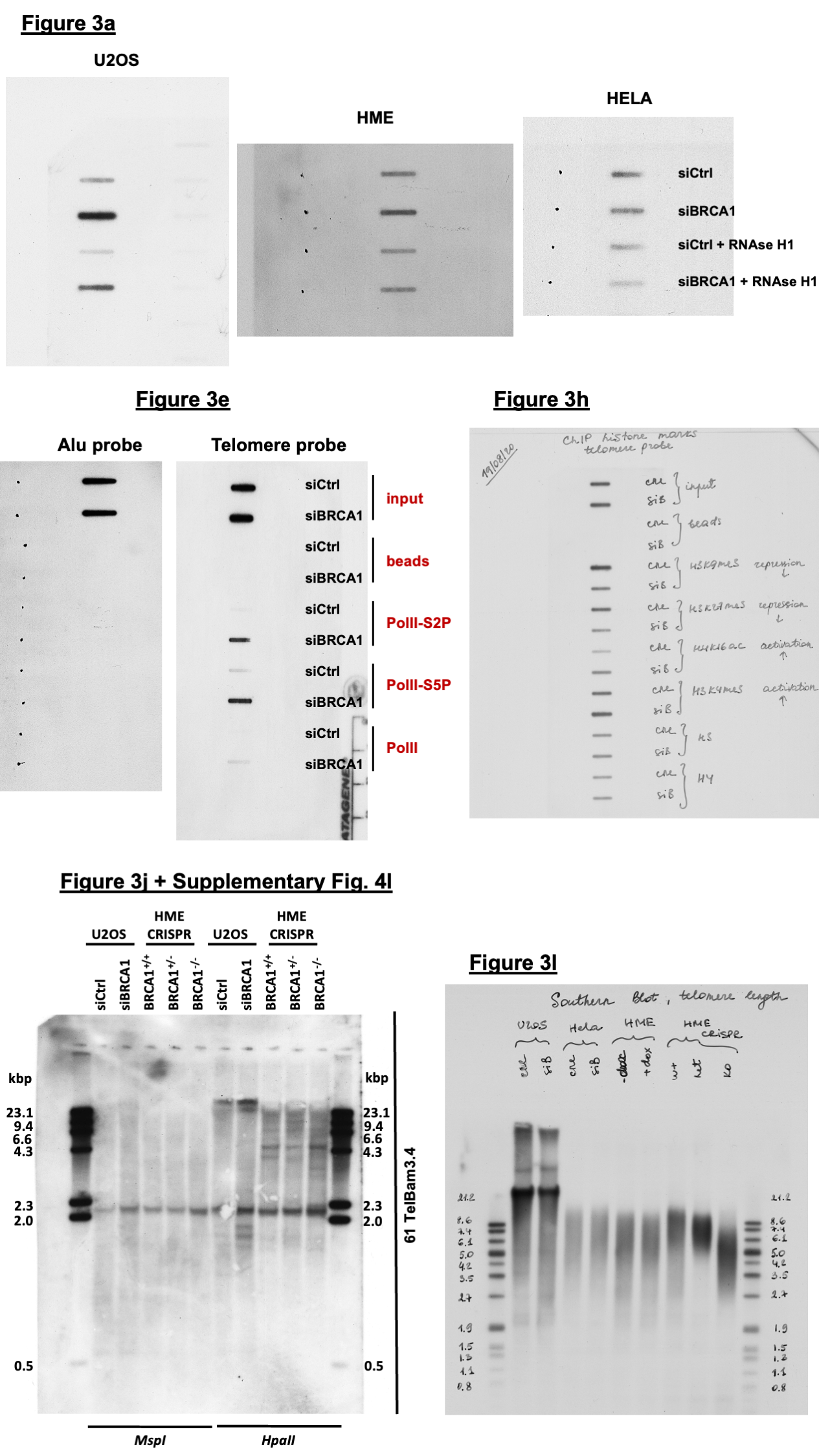

Supplement: Supplementary file 4 — Source Data [file 41467_2021_23716_MOESM4_ESM.zip › 266613_2_supp_5525016_qs7406.png]

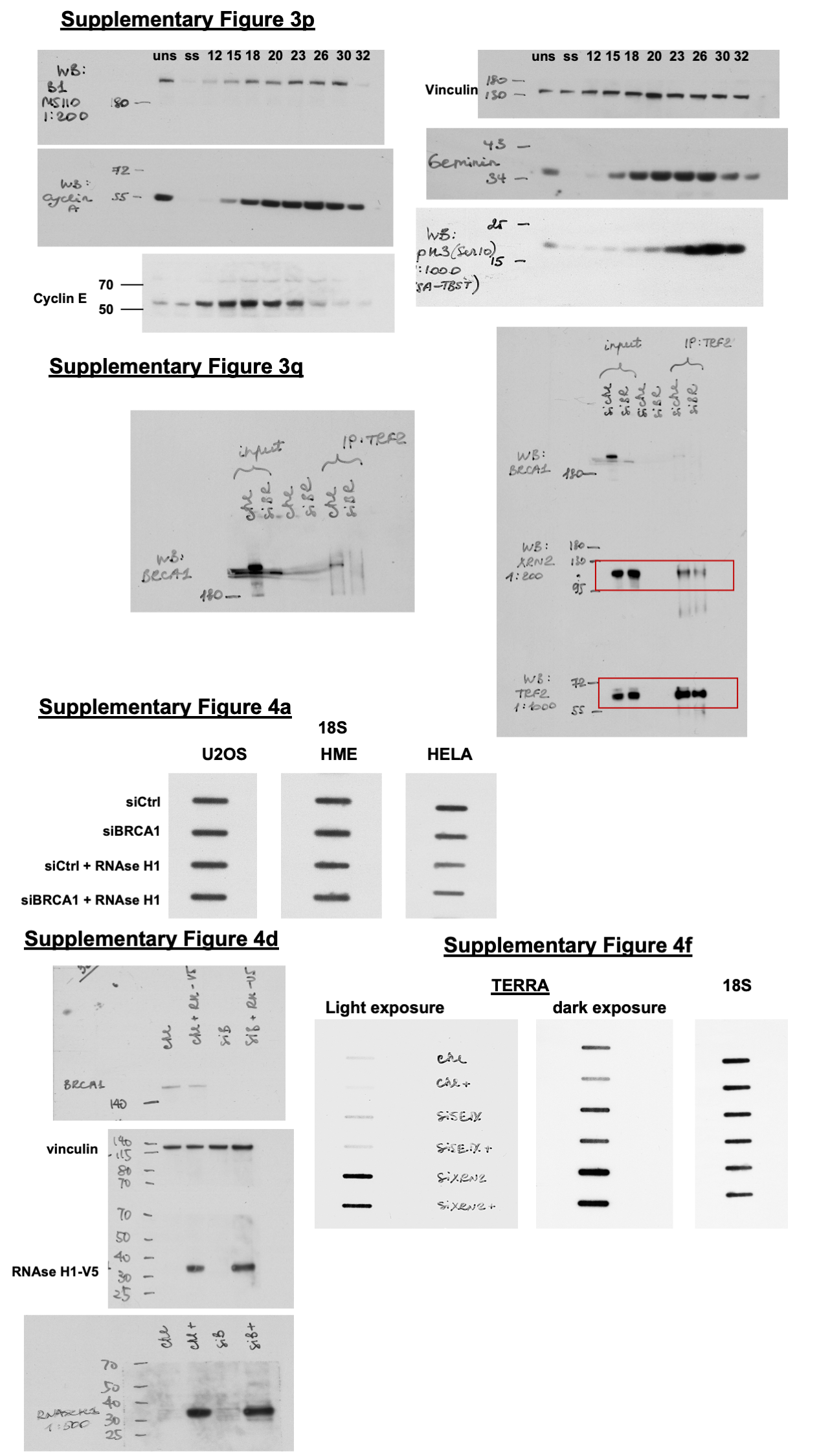

Supplement: Supplementary file 4 — Source Data [file 41467_2021_23716_MOESM4_ESM.zip › 266613_2_supp_5525017_qs7406.png]

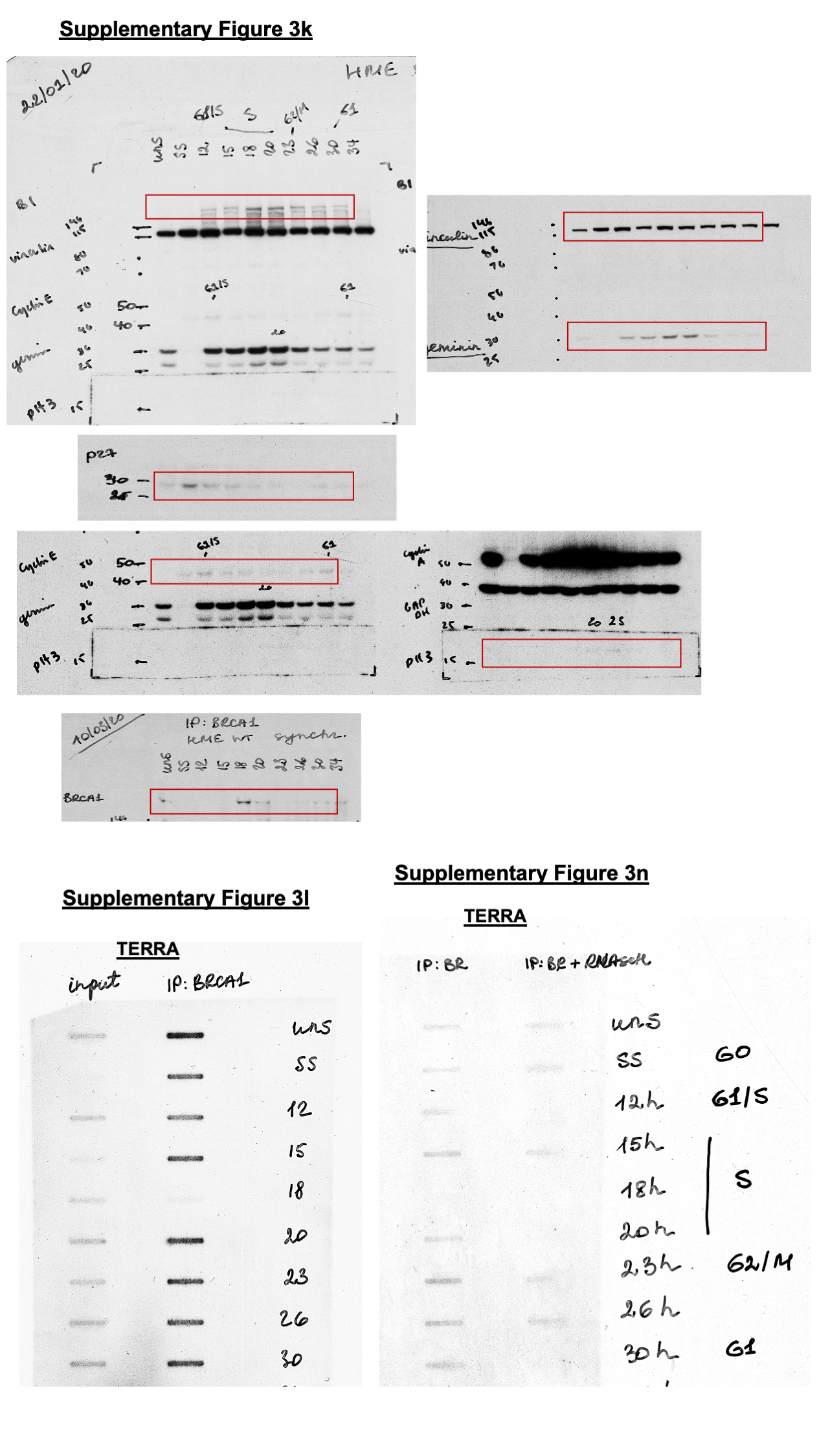

Supplement: Supplementary file 4 — Source Data [file 41467_2021_23716_MOESM4_ESM.zip › 266613_2_supp_5525018_qs7406.png]

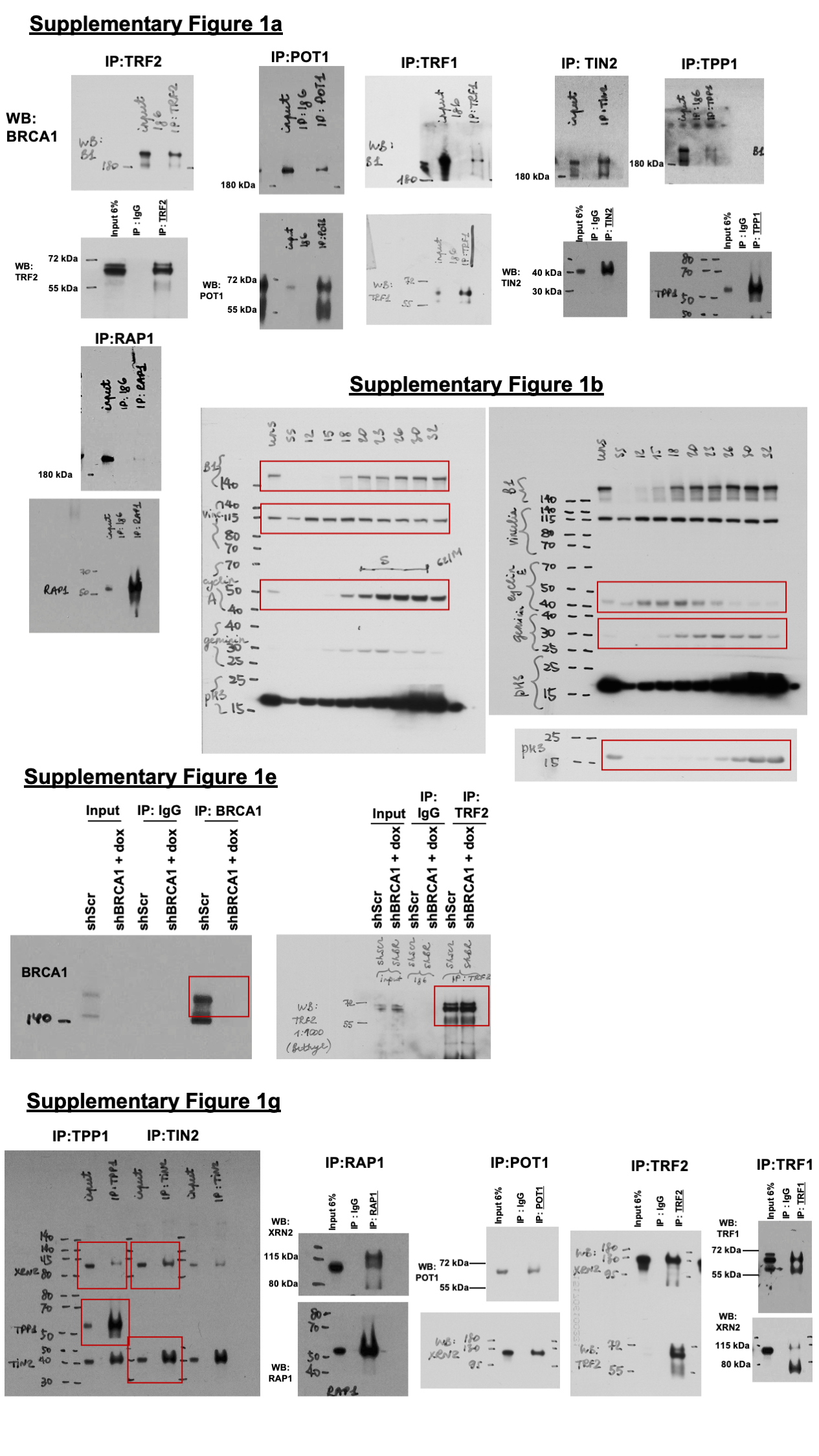

Supplement: Supplementary file 4 — Source Data [file 41467_2021_23716_MOESM4_ESM.zip › 266613_2_supp_5525006_qs7405.png]

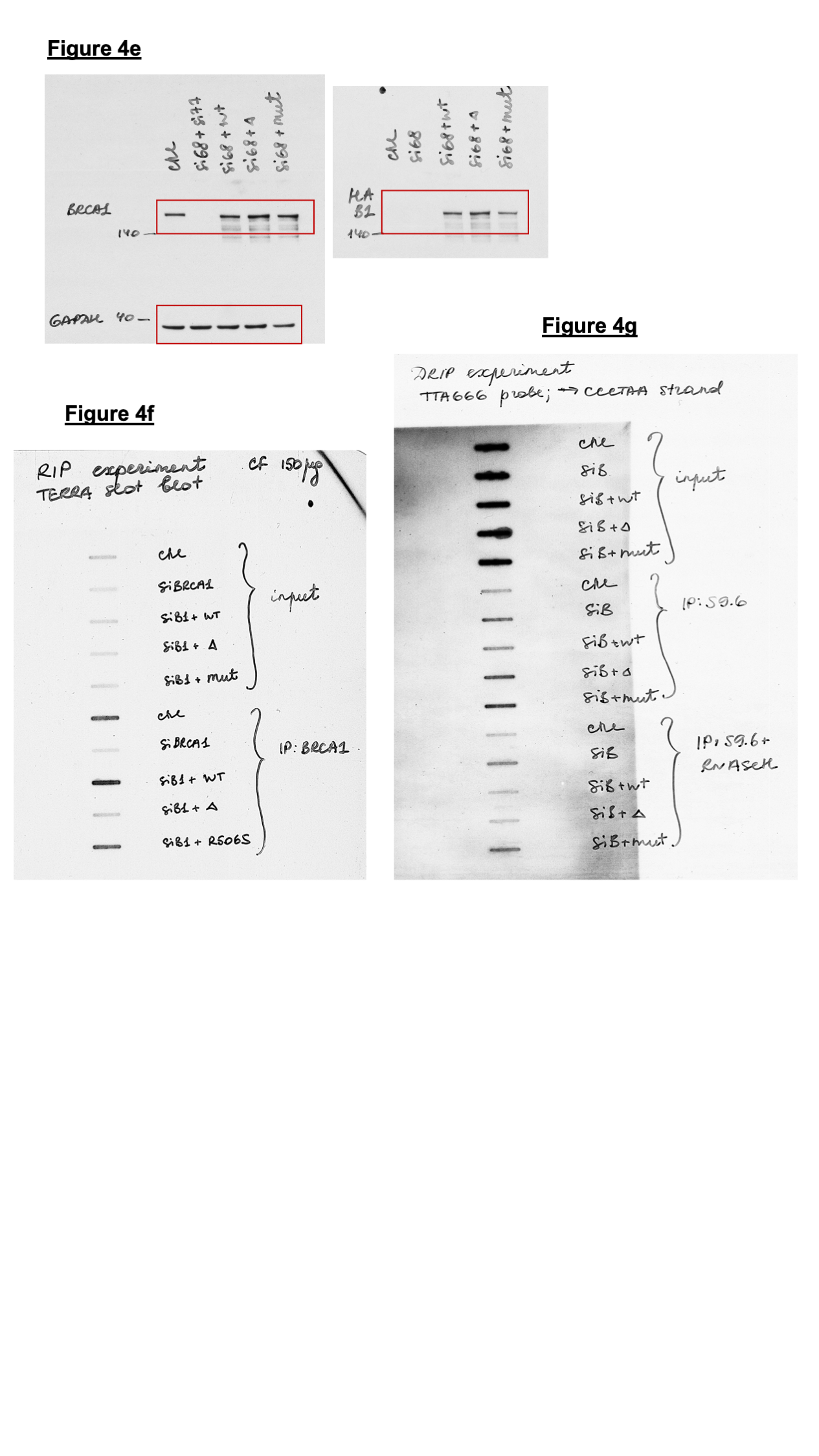

Supplement: Supplementary file 4 — Source Data [file 41467_2021_23716_MOESM4_ESM.zip › 266613_2_supp_5525007_qs7405.png]

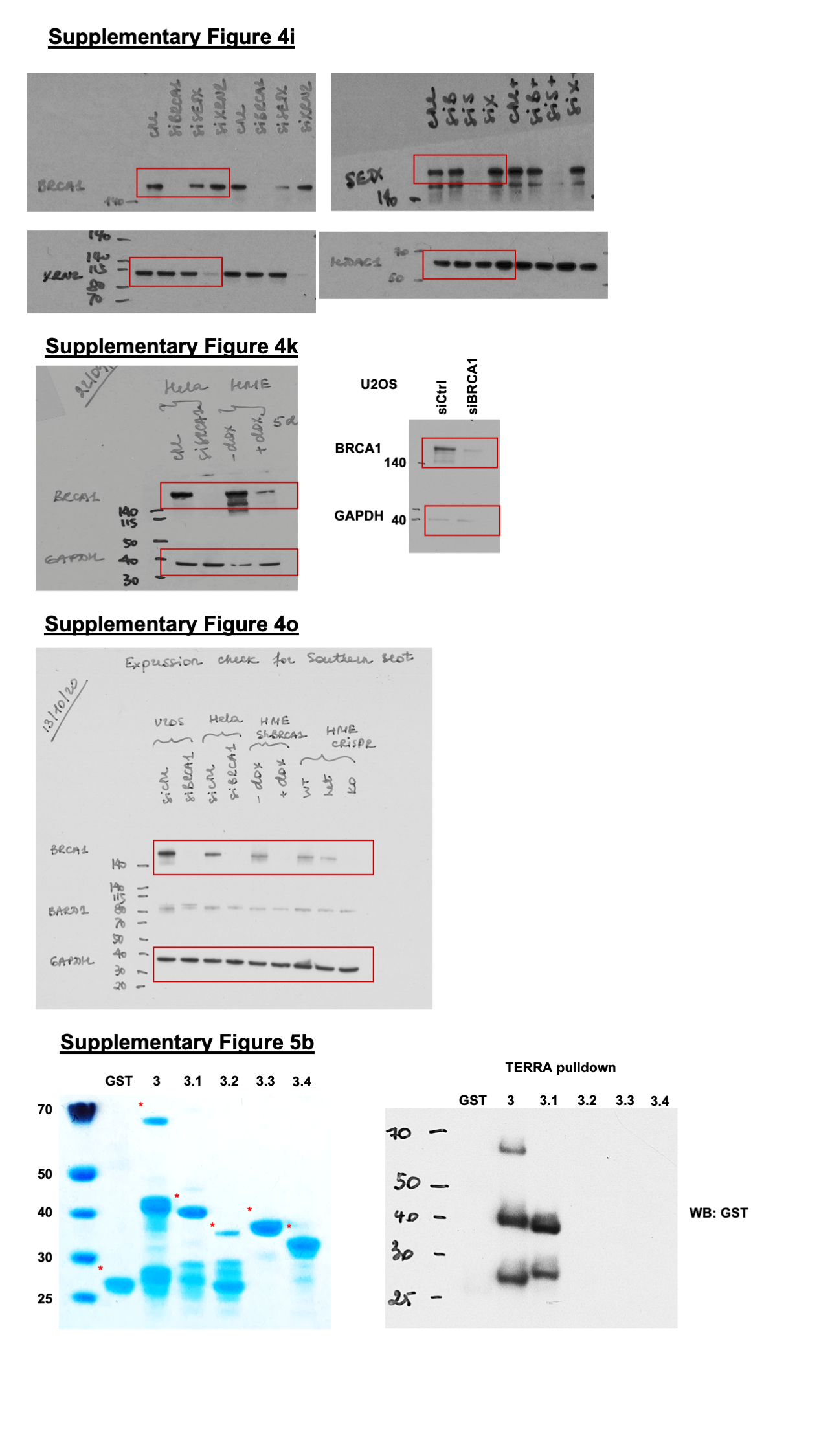

Supplement: Supplementary file 4 — Source Data [file 41467_2021_23716_MOESM4_ESM.zip › 266613_2_supp_5525008_qs7405.png]
